# Reconstruction of cell diversity and cell lineages from somatic mutations in single-cell transcriptomic data

**DOI:** 10.1093/dnares/dsag007

**Published:** 2026-08-25

**Authors:** Satoshi Oota, Kuniya Abe, Cheng-Tsung Pan, Hideo Yokota, Wen-Hsiung Li, Kazuho Ikeo

**Affiliations:** Center for Advanced Photonics, RIKEN, Wako, Saitama 351-0198, Japan; BioResource Research Center, RIKEN, Tsukuba, Ibaraki 305-0074, Japan; Graduate School of Integrated Sciences for Life, Hiroshima University, Higashi-Hiroshima, Hiroshima 739-0046, Japan; Laboratory for Regulatory Genomics, RIKEN Center for Integrative Medical Sciences, Yokohama, Kanagawa 230-0045, Japan; Center for Advanced Photonics, RIKEN, Wako, Saitama 351-0198, Japan; Biodiversity Research Center, Academia Sinica, Taipei 11529, Taiwan; Department of Ecology and Evolution, University of Chicago, Chicago, IL 60637, United States; DNA Data Analysis Laboratory, National Institute of Genetics, Mishima, Shizuoka 411-8540, Japan

**Keywords:** somatic mutations, phylogenetic tree, scRNA-seq data, normal tissue

## Abstract

Single-cell RNA sequencing (scRNA-seq) enables high-resolution profiling of individual cells. However, inferring temporal relationships among cells remains a challenge. Here, we present real-time course analysis (RTCA), a simple direct phylogenetic signal framework that reconstructs cell lineages from somatic variants detected across cells using variants derived from nuclear-encoded transcripts in scRNA-seq data. Our simulations demonstrate that only a modest increase in informative variant sites is sufficient to maintain accurate tree reconstruction, even with a tenfold increase in the number of cells. This scalability makes RTCA applicable to datasets generated by recent single-cell sequencing technologies and is also suitable for reanalyzing existing datasets. We also performed a comparative analysis between our method and a representative genotype-mediated inference framework, PhylinSic, which shares certain conceptual similarities with RTCA. The simulation results show that RTCA is more robust to sparse mutation signals and dropout-induced missing data than PhylinSic. In an application to the datasets from 2 *healthy* human placental samples, RTCA successfully reconstructed bifurcating phylogenetic trees. By mapping expression-based cell type clusters onto the trees, we evaluated the degree of monophyly within lineages and found our results consistent with known placental differentiation pathways. The identified cell lineages also aligned with classifications based on gene expression and pseudotime analysis. Compared to gene expression-based and pseudotime analysis, RTCA provides a temporally based model of cell trajectories, integrating lineage and expression information in a biologically meaningful manner. In summary, RTCA offers a scalable, cost-effective solution for reconstructing developmental processes in complex normal tissues.

## 1. Introduction

Somatic mutations accumulate during development and ageing,^1^ resulting in cellular mosaicism in somatic tissues and are associated with various diseases, including dementia, cardiovascular diseases, and cancers.^2,3^ In some cases, somatic mutations contribute to normal development, such as the acquisition of the diversity of neuronal cell populations in the central nervous system.^4^ Furthermore, a large-scale RNA sequencing revealed that clonal expansions of somatic mutations occur across diverse normal tissues.^5^

It has been estimated that during early human development, 2.8 somatic mutations occur per cell per replication event (95% confidence interval: 2.4 to 3.3), which is slightly higher than the rate observed for germline mutations.^6^ In later life, somatic mutation rates range from 6.4 × 10^−10^ to 7.6 × 10^−10^ mutations per base pair per cell division across various cell types.^7^ These rates can reach up to 10 times higher than those observed in the germline.^8^ Recent studies indicate that the best-supported upper range is approximately 1 to 8 single-nucleotide variants (SNVs) per genome per cell division (corresponding to ∼0.3 to 2.7 × 10^−9^ mutations per base per division).^9,10^ As a consequence, even monozygotic twins can exhibit detectable genetic differences due to post-zygotic somatic mutations.^11^

Somatic mutations are ubiquitous across a wide range of organisms, including humans,^12^ and can be regarded as counterparts to germline mutations,^10^ both of which are subject to evolutionary forces.^13^ Similar to germline cells, each somatic cell carries an “evolutionary record” in the form of mutational “scars” that accumulate over time in the genome. Therefore, it is possible to computationally reconstruct a cell's history using a retrospective lineage tracing approach,^14,15^ as somatic mutations inherently encode temporal information about the cell's lineage.

Pioneered by Charles Whitman^16^ in the 19th century for the developmental biology of invertebrates, lineage tracing is now an essential tool to study stem cells in adult mammalian tissues. Somatic mutations have been extensively studied in the context of cancer evolution.^17^ Researchers have devised mathematical models to elucidate somatic mutation dynamics from various biological perspectives.^18^

Single-cell sequencing enables a wide range of applications, including the investigation of rare cell types, uncultured microbes, and somatic tissue mosaicism.^19^ While detecting somatic mutations through genome sequencing is conceptually direct—since these mutations arise at the DNA level—single-cell genome sequencing (scDNA-seq) is constrained by a critical limitation: each cell harbours only 2 copies of each DNA molecule. This extreme scarcity of input material poses major technical hurdles, such as coverage nonuniformity, allelic dropout (ADO), and elevated rates of false positives and false negatives.^20^

In contrast, single-cell RNA sequencing (scRNA-seq) offers several practical and technical advantages. The presence of hundreds to thousands of RNA copies per gene substantially boosts the molecular input available for analysis, reducing stochastic dropout and enhancing the signal-to-noise ratio. Moreover, the overwhelming abundance and accessibility of scRNA-seq datasets provide a powerful platform for high-throughput somatic variant discovery across diverse biological systems.^21^

Although challenges such as sparse coverage in lowly expressed genes,^22^ ADO,^23^ amplification bias,^24^ RNA editing,^25^ and technical noise^26^ persist, these limitations are increasingly being addressed through innovative computational and experimental strategies. Expression bias, once a major hurdle, can now be accounted for using statistical models, and integrative approaches that leverage transcriptomic context allow for more refined mutation calling even in the absence of matched DNA.^27^

Thus, while scRNA-seq was not originally designed for mutation analysis, its scalability, molecular redundancy, and growing methodological support make it a compelling and increasingly preferred alternative to scDNA-seq for the detection of somatic mutations—especially in studies aiming to link genotype with gene expression at single-cell resolution.^28^

Many methods have been proposed to reconstruct cell lineage trees using endogenous mutations detected in scRNA-seq data. PhylinSic^29^ employed a probabilistic smoothing strategy to denoise variant calls before inferring a Bayesian phylogeny, providing a complete mutation-to-tree pipeline based on scRNA-seq data from cancer cells. Moravec et al.^30^ introduced a novel approach to cancer evolutionary studies by demonstrating that scRNA-seq data contains sufficient phylogenetic signal for reconstructing the population history of cancer cells. LINEAGE^31^ took a label-free approach, identifying endogenous markers—primarily mitochondrial RNA mutations—from standard scRNA-seq to infer lineage relationships without engineered barcodes. Although the study focused on mitochondrial variants, it illustrated the feasibility of an integrated scRNA-only workflow. Other tools, such as mgatk^32^ and MQuad,^33^ identified informative mtDNA variants in scRNA-seq and related assays to enable clonal and lineage inferences. More recent pipelines (eg, MitoTracer,^34^ MitoTrace,^35^ scMitoMut^36^) extended this strategy, while studies such as that of Ludwig and Lareau^32^ demonstrated that somatic mtDNA mutations captured in scRNA-seq can serve as lineage markers while simultaneously preserving information on cell state.

Another line of work integrated DNA and scRNA-seq to jointly reconstruct clonal or lineage trees. Cardelino^37^ links scRNA-seq cells to clones inferred from bulk exome sequencing, while PhylEx^38^ explicitly combined bulk DNA-seq with scRNA-seq to recover accurate clonal trees. Similarly, MaCroDNA^39^ and clonealign^40^ align scRNA-seq profiled clones inferred from scDNA or bulk sequencing, providing more robust lineage reconstruction when mutation signals in scRNA-seq alone are too sparse.

However, these integrative approaches—including the scRNA-only pipelines described above—have been developed and validated primarily in cancer tissues, where the mutation burden is approximately 4 to 100 times higher than in normal tissues.^9^ As a result, robust frameworks for reconstructing lineage trees from nuclear SNVs detected solely in scRNA-seq data from normal tissues remain scarce. This reflects the prevailing assumption that normal cells accumulate too few somatic mutations^41^ to yield sufficient phylogenetic signal.

Here, we propose a simple direct phylogenetic signal framework, termed *Real-Time Course Analysis* (RTCA), for inferring cell lineage trajectories during the development of normal tissues or organs using scRNA-seq data alone. Broadly speaking, RTCA belongs to the class of retrospective cell-tracing approaches that exploit somatic variations in single-cell genomes. However, RTCA possesses a nuanced yet distinctive characteristic compared with existing lineage-inference methods based solely on nuclear and/or mitochondrial scRNA-seq data, such as PhylinSic^29^ and LINEAGE.^31^ These methods are based on phylogenetic frameworks in which lineage inference is mediated through latent per-cell genotype representations,^42–44^ in which lineage inference is mediated through per-cell genotype representations, whereas RTCA is based on a direct phylogenetic signal framework, accumulating phylogenetic signal across cells without introducing an explicit per-cell genotype layer (for details, see Materials and Methods and Discussion).

In other words, RTCA directly leverages phylogenetic signals embedded within scRNA-seq data across cells, without requiring additional information. By contrast, most existing approaches are implicitly grounded in a cladistic framework based on per-cell genotypes—focusing primarily on character presence–absence patterns—rather than on an explicit phylogenetic framework that models genuine evolutionary processes.^29,30,45,46^

RTCA also serves as a counterpart to dimension reduction methods used for categorizing cell types and performing pseudotime analysis based on unsupervised machine learning approaches, such as t-distributed stochastic neighbour embedding (t-SNE)^47^ and uniform manifold approximation and projection (UMAP).^48^ These methods sometimes lack both biologically relevant interpretations and reproducibility of global data structure.^49^

To evaluate the performance of our method, we conducted simulations to evaluate the power of our method to reconstruct accurate bifurcating cell lineage trees from a relatively small number of variant sites. In this simulation, we generated sequence data by extracting variant sites from randomly constructed sequences. The accuracy of lineage inference depends on several factors, including the number of variant sites and the sparsity of variant calls. Generally speaking, longer sequences have more variant sites, providing richer phylogenetic information than shorter sequences. Similarly, higher diversity can enhance phylogenetic resolution, but excessive diversity may obscure underlying lineage signals.^50^

We also conducted a set of simulations incorporating scRNA-seq–specific noise, in which stochastic errors were introduced according to empirically motivated per-base error rates.

As a proof-of-concept, we reconstructed lineage trees of normal placental tissue cells (Figure S1), using single-cell somatic variants obtained from high-quality scRNA-seq data. Moreover, we integrated the somatic variant-based cell trajectories with the corresponding gene expression profiles. This application demonstrated RTCA’s ability to reconstruct biologically meaningful, temporally resolved cell trajectories that unify lineage relationships with gene expression profiles. It highlights RTCA’s potential as a scalable and cost-efficient framework for decoding developmental dynamics in complex tissues using transcriptome-derived somatic variants.

## 2. Materials and methods

### 2.1. Expected number of observable segregating sites

The expected number of segregating sites in a sequence depends on the number of unique mutations, rather than the total number of mutational events. Under the infinite-sites model (ie, no recurrent mutations), the expected number of segregating sites is approximately equal to the number of unique mutations^51–54^:


(1) E[S]≈(Totalnumberofcelldivisions)×L×μ,

where *L* is the length of the sequence (eg, $3\times 10^{9}$ bp for the human genome), and *μ* is the somatic mutation rate per base per cell division.

We assume that the human placenta contains approximately $5\times 10^{11}$ terminal cells (ie fully differentiated or endpoint cells).^55,56^ Under the simplifying assumption that each such terminal cell corresponds to a unique cell-division event (ie no convergent lineages), the upper bound on the total number of divisions in the placental lineage is therefore $5\times 10^{11}$.

Under the infinite-sites model, and assuming that all resulting mutations are detectable, the expected number of segregating sites for a genome is


(2) $$E[S{]}_{\mathrm{genome}}=5\times 10^{11}\times 3\times 10^{9}\times \mu .$$

For the lower bound ($\mu =0.3\times 10^{-9}$)^10,12,57^: $E[S{]}_{\mathrm{genome}}=$  $4.5\times 10^{11},$ and for the upper bound ($\mu =2.73\times 10^{-9}$)^9,10,57^: $E[S{]}_{\mathrm{genome}}=4.05\times 10^{12}.$

Because scRNA-seq covers only a subset of the genome, the effective number of segregating sites in transcribed regions (targets) must be scaled down accordingly. Let $L_{\mathrm{target}}$ denote the effective size of the expressed (transcribed) region and $L_{\mathrm{genome}}=3\times 10^{9}$. Then,


(3) $$E[S{]}_{\mathrm{target}}=E[S{]}_{\mathrm{genome}}\times \frac{L_{\mathrm{target}}}{L_{\mathrm{genome}}}.\;$$

We make the following assumptions:

- For exome: $L_{\mathrm{exome}}=30\mathrm{Mb}$; hence $L_{\mathrm{exome}}/L_{\mathrm{genome}}=1/100$ and $E[S{]}_{\mathrm{exome}}\approx 4.5\times 10^{9}\text{–}4.05\times 10^{10}$.^58,59^
- For transcribed regions (TX): $L_{\mathrm{TX}}=50-100\mathrm{Mb}$; hence $L_{\mathrm{TX}}/L_{\mathrm{genome}}=1/60-1/30$ and that $E[S{]}_{\mathrm{TX}}\approx 7.5\times 10^{9}\text{–}$  $1.35\times 10^{11}$.^60,61^

Based on reported estimates of turnover in human placental trophoblast populations,^62–66^ we adopt a working assumption of ∼3 × 10^13^ total mitoses in the placenta over gestation (see Supplementary material for detail). With a final surviving cell count of ∼5 × 10^11^, this yields a minimum doubling number of $\mathrm{lo}g_{2}(5\times 10^{11}\times L_{\mathrm{target}}/L_{\mathrm{genome}})=$  $\mathrm{lo}g_{2}(5\times 10^{11}/30)\approx 34$ and an implied lineage turnover factor of $∼(3\times 10^{13}/(5\times 10^{11}))=∼60$.

Therefore, the cumulative lineage depth per terminal cell^62^ is h≈34×60=2,040divisionspertip. The *h* represents an estimate of the cumulative mitotic activity per surviving lineage—that is, the total number of cell divisions contributing, directly or indirectly, to each extant cell when accounting for population-level turnover. This value should not be interpreted as the number of sequential mitoses experienced by a single lineage, which is expected to be in the range of tens to low hundreds. For *n* sampled cells, the total tree length in terms of “divisions” is roughly


(4) $$E[T_{n}]\;\approx \;nhH_{n-1},$$

where $H_{n-1}=\overset{n-1}{\underset{k=1}{\sum }}\;\frac{1}{k}$ is the (n−1)-th harmonic number.^67,68^

Thus, for n=1,000 ($H_{999}\approx 7.484$), $E[T_{1000}]\approx$  $1,000\times 2,040\;\times \;7.484\;\approx 1.51\times 10^{7}\;\mathrm{divisions}.$

Under the infinite sites model,


(5) $$E[S]\;=\;\;E[T_{n}]\times L_{\mathrm{target}}\times \mu .$$

However, only a fraction of variants are detectable in scRNA-seq data due to technical limitations such as incomplete transcript capture,^26^ monoallelic expression and ADO,^69^ limited read coverage,^70^ and the sensitivity of variant callers.^71^

Let the fraction of detectable variants be


(6) $$f_{\mathrm{detectable}}=f_{\mathrm{expr}}\cdot f_{\mathrm{allele}}\cdot f_{\mathrm{coverage}}\cdot f_{\mathrm{caller}},$$

where $f_{\mathrm{expr}}$ denotes the transcript detection rate (reflecting dropout), $f_{\mathrm{allele}}$ the allelic expression rate (affected by monoallelic expression or ADO), $f_{\mathrm{coverage}}$ the read coverage and abundance filtering, and $f_{\mathrm{caller}}$ the variant-calling sensitivity in scRNA-seq.

Representative detectability factors were set to $f_{\mathrm{expr}}=0.2$, $f_{\mathrm{allele}}=0.6$, $f_{\mathrm{coverage}}=0.7$, and $f_{\mathrm{caller}}=0.3$ (Table 1), based on the published benchmarks of transcript detection,^26,70^ allelic expression,^69^ coverage and sequencing depth,^70^ and variant-calling sensitivity in scRNA-seq.^71^ Then, the overall $f_{\mathrm{detectable}}$ can be calculated as 0.2×0.6×0.7×0.3=0.0252. Therefore, in the case of exome scale ($L_{\mathrm{target}}=3\times 10^{7}$ bp), it follows from Eq. (5) that for $E[T_{1000}]\approx 2.73\times 10^{5}\;\mathrm{divisions}$  $E[S]\approx (0.3\times 10^{-9}\;\mathrm{to}\;2.7\times 10^{-9})\times 3\times 10^{7}\times 1.51\times 10^{7}\approx$  $1.36\times 10^{5}\;\mathrm{to}\;1.22\times 10^{6}.$

**Table 1.** Representative detectability factors.

Factor	Symbol	Typical Range	Effect
1. Transcript detection rate (dropout)^26,70^	$f_{\mathrm{expr}}$	0.05 to 0.5	Fraction of mutations that are present in transcripts and are captured in sequencing
2. Allelic expression rate (monoallelic or allelic dropout)^69^	$f_{\mathrm{allele}}$	0.5 to 1.0	Chance that the mutated allele is expressed
3. Read coverage and abundance filter^70^	$f_{\mathrm{coverage}}$	0.5 to 0.9	Mutations need ≥1 to 3 reads to be called
4. Variant calling sensitivity in scRNA-seq^71^	$f_{\mathrm{caller}}$	0.1 to 0.5	Variant caller sensitivity (RNA-Mutect2^5,72^ etc.)

Note: scRNA-seq alignment data typically contain approximately 10^−3^ erroneous mismatches per base (0.1%),^26^ along with allelic dropout affecting 20 to 70% of heterozygous loci^70^ and RNA-editing events occurring at rates of approximately 10^−5^ to 10^−4^ per base.^73^

From Eq. (4), $E[T_{1000}]$ can be calculated as $2.73\times 10^{5}$ divisions. $E[S{]}_{\mathrm{exome}}$ can be calculated as $(0.3\times 10^{-9}\;\mathrm{to}\;2.7\times 10^{-9})\times 3\times 10^{7}\times 1.51\times 10^{7}1.36\times 10^{5}\;\mathrm{to}\;\;1.22\times 10^{6}$.

The human exome, including only protein-coding regions, has been estimated to span approximately 30 Mb, corresponding to about 1% of the genome.^58^ In contrast, the transcribed (tx) portion of the genome covers roughly 50 to 100 Mb.^60,74^ Therefore, scaling the exome-based estimate by a factor of 50/30 to 100/30 yields $E[S{]}_{\mathrm{tx}}\approx 2.27\times$  $10^{5}\text{–}4.53\times 10^{5}.$

With $f_{\mathrm{detectable}}=0.0252$, the number of detectable segregating sites in scRNA-seq is


$$E[S{]}_{\mathrm{detectable},\mathrm{exome}}\approx 3.42\times 10^{3}\text{–}3.08\times 10^{4},$$

or


$$E[S{]}_{\mathrm{detectable},\mathrm{tx}}\approx 8.56\times 10^{3}\text{–}7.71\times 10^{4}.$$

Hence, under theoretical expectations, normal tissues should harbour a substantial number of segregating sites. The next question is whether the expected number of segregating sites is sufficient to reconstruct a reliable binary tree.

### 2.2. Relationship between sample size and expected branch length

When we reconstruct a phylogenetic tree from segregating sites, we use only mutations that are segregating in the sampled cells (ie, polymorphic positions), as fixed or invariant sites contain no phylogenetic information.^51,52^

Fixed sites (ancestral or derived) are excluded from phylogenetic reconstruction; thus, branch lengths in the resulting tree reflect only the number of *informative* (segregating) sites shared among lineages, rather than the total number of divisions or absolute mutation burden.^45,75^

From the coalescent theory under the neutral, infinite sites model, the expected number of segregating sites among *n* sampled lineages is


(7) $$E[S]=\theta H_{n-1},$$

where $\theta =4N_{e}\mu L$ in classical population genetics,^51,52,76^ or, for somatic cell lineages, it can be approximated by θ≈2μLh, in which the cumulative lineage depth *h* replaces $2N_{e}$.^77,78^ Here, $H_{n-1}=\overset{n-1}{\underset{k=1}{\sum }}\;\frac{1}{k}\approx \ln(n)+\gamma$ is the harmonic number.

Under the neutral coalescent, the expected total tree length in units of generations is


(8) $$E[T_{n}]=2h\times H_{n-1}.$$

The total number of segregating sites is proportional to the total tree length ($E[S]=\mu LE[T_{n}]$,^51,75^) and hence, the average branch length reflects the expected number of informative sites per lineage segment rather than the total number of cell divisions^77^:


(9) $$\mathrm{Average}\;\mathrm{branch}\;\mathrm{length}=\frac{E[S]}{\mathrm{Number}\;\mathrm{of}\;\mathrm{branches}}.$$

For a rooted binary tree of *n* taxa, there are 2n−2 branches.^45^

Thus, the average branch length can be expressed as


(10) $$\bar{b}=\frac{E[S]}{2n-2}.$$

Substituting $E[S]=\mu LE[T_{n}]$ and $E[T_{n}]=2hH_{n-1}$,^51,68,76^ we obtain


(11) $$\bar{b}=\mu L\frac{hH_{n-1}}{n-1},$$

showing that the expected branch length is proportional to the total number of segregating mutations per lineage segment.

With 1,000 sampled cells from the placenta, under our assumptions (Table 2), the expected average branch length is given by $\bar{b}=\mu L\frac{hH_{n-1}}{n-1}=\mu (3\times 10^{7})\frac{2,040\times 7.48}{999}.$

**Table 2.** Summary of parameters for placenta data.

Parameter	Symbol	Value
Mutation rate	*μ*	$0.3\times 10^{-9}$ − $2.7\times 10^{-9}$^9,10^
Target region	*L*	$3\times 10^{7}\;\mathrm{bp}\;$ (exome)
Effective divisions per tip	*h*	2,040
Sample size	*n*	1,000
Harmonic number	$H_{999}\approx 6.9+0.577=7.48$	

For the lower ($\mu =0.3\times 10^{-9}$) and upper ($\mu =2.7\times 10^{-9}$) bounds, $\bar{b}=0.3\times 10^{-9}\times 4.58\times 10^{8}\approx 0.14$ and 1.23, respectively.

For the lower and upper bounds, $\bar{b}$ can be calculated as 0.14 and 1.23 using $\mu =0.3\times 10^{-9}$ and $2.7\times 10^{-9}$, respectively. Therefore, the average branch length is roughly 0.1 to 1.2 segregating sites per branch.

**Fig. 1.**
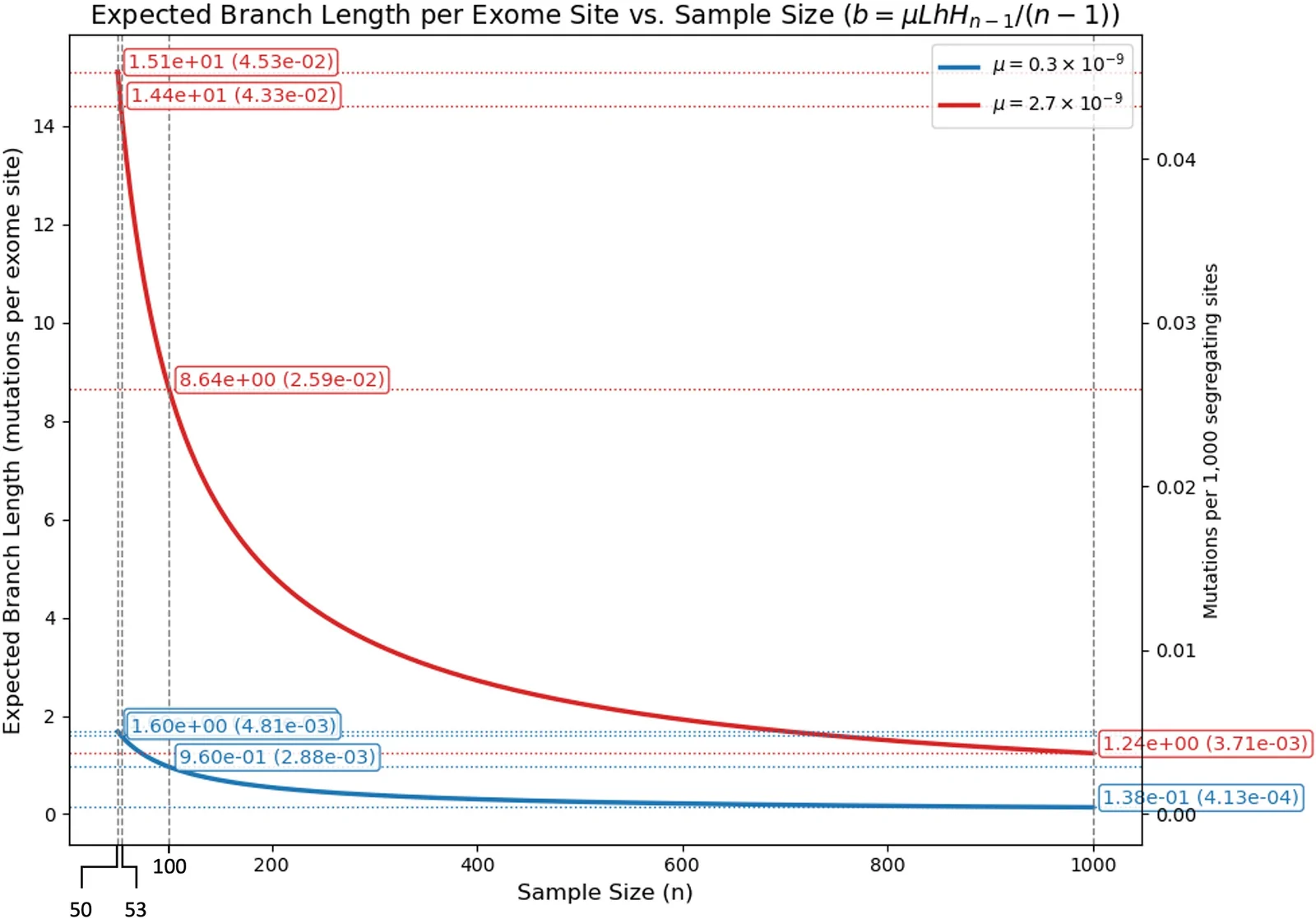
Expected branch length per exome site as a function of sample size. The left *y*-axis represents the expected branch length in units of mutations per exome site, while the right *y*-axis shows the corresponding values scaled to mutations per 1,000 segregating sites. The *x*-axis indicates the number of sampled cells (*n*, from 50 to 1,000). The 2 curves correspond to different somatic mutation rates: a lower bound ($\mu \;=\;0.3\;\times \;10^{-9}$, blue) and an upper bound ($\mu \;=\;2.7\;\times \;10^{-9}$, red). Estimates assume a target region of $L=3\times 10^{7}$ bases (exome) and an effective cumulative mitotic depth of $\mu \;=\;2.7\;\times \;10^{-9}$ cell divisions per terminal cell (see Table 2 for parameters). Vertical dashed lines mark representative sample sizes (n=50,53,100,and1,000). The dashed line at n=53 corresponds to the sample size used in the Batch 1 real-data analysis, thereby linking the theoretical expectation shown here with the empirical analyses presented in the Materials and Methods, Results, and Discussion sections. Dotted horizontal lines indicate the corresponding branch length values for each mutation rate. The intersection labels show the predicted branch lengths in both scales, illustrating how increasing sample size reduces the average branch length due to the dilution of shared mutations among more lineages. Values in parentheses indicate mutations per 1,000 segregating sites per branch (right axis).

Figure 1 is the plot of expected average branch length vs. sample size (*n*), based on 2 mutation rates (low: $\mu =0.3\times 10^{-9}$ and high $\mu =2.7\times 10^{-9}$), target region $L=3\times 10^{7}$ bases (eg, exome), and effective depth h=2,040 divisions per surviving cell. Vertical lines mark the commonly analyzed sample sizes: *n* = 50, 100, and 1,000.

### 2.3. Formalization of variant patterns in scRNA-seq data

We now formalize the detection of somatic mutations in scRNA-seq data using first-order predicate logic symbols.^79–81^ This approach follows earlier studies that applied formal logic and ontology frameworks to biological data representation and reasoning.^82,83^ The primary objective of this formalization is to establish a well-defined framework for implementing detection algorithms while maintaining flexibility to accommodate the rapid evolution of scRNA-seq technologies. As the detailed formulation of the first-order logic is lengthy, we provide only a concise explanation here; further details are available in the Supplementary material.

A somatic mutation is expected to be present only in a subset of cells. Formally, we define:

-
Som(m) denotes that mutation *m* is a somatic mutation; and
-
O(c,a) denotes that in cell *c*, the allele type *a* is observed, where *a* ∈ {R, V}, representing the reference (R) and variant (V) alleles.

Then, the logical condition for a somatic mutation can be expressed as:


(12) $$\begin{array}{rl} & \mathrm{Som}(m)\to (\exists c_{1}[O(c_{1},V)\wedge \neg O(c_{1},R)]\wedge \exists c_{2}[O(c_{2},R)\wedge \\ & \neg O(c_{2},V)\wedge c_{1}\neq c_{2}]), \end{array}$$

where ∃, ∧, and ¬ denote “there exists,” “and,” and “not,” respectively.

In other words, if *m* is a somatic mutation, there exists at least one cell ($c_{1}$) where the variant is observed but the reference is not, and at least one distinct cell ($c_{2}$) where the reference is observed but the variant is not.

In a mosaic scenario (genetic mosaicism),^84^ at least one cell shows evidence of the variant while at least another does not. In contrast, for a germline mutation (whether inherited or de novo in the germline), the variant would be observed consistently across all cells, aside from occasional ADOs (missing expression due to stochastic transcriptional variability).

To distinguish somatic from germline variants, we perform cross-cell comparisons—ie, we consider variant calls across all cells.

- Germline:
  (13) $$\begin{array}{rl} & \forall c,\;\;g(c)\in \{R,V\}\;\;(\mathrm{The}\;\mathrm{variant}\;\mathrm{appears}\;\mathrm{in}\;\mathrm{every}\;\mathrm{cell},\\ & \mathrm{except}\;\mathrm{for}\;\mathrm{dropouts}.) \end{array}$$
- Somatic:
  (14) $$\begin{array}{rl} & \exists \;c_{1},\;g(c_{1})=V\;\text{and}\;\exists \;c_{2},\;g(c_{2})=R\\ & \;\text{(at least one cell carries the variant,}\\ & \mathrm{while}\;\mathrm{another}\;\mathrm{retains}\;\mathrm{the}\;\mathrm{reference}). \end{array}$$

With this formalization, we can propose the following theorem, which is readily provable under the infinite-sites model^51–54^:

### 2.4. Theorem

Under the assumption that at most one new (de novo) mutation can occur per site in the germline, it follows that at least one somatic mutation must have occurred at any tri-allelic variant site (see Materials for the proof of the theorem). Below, we will apply this theorem to identify as many partial somatic mutations as possible in our scRNA-seq data.

### 2.5. Simulation for evaluating cell lineage tree reconstruction accuracy

The simulation consists of the following steps:

1. **Random tree generation:** A binary tree is generated with a given average branch length using the generate_random_tree() function in the Environment for Tree Exploration (ETE) v3.^85^ This tree serves as the true tree for evaluating tree reconstruction accuracy. The average branch length was set to the reciprocal of the sequence length, ensuring that each variant site is expected to have at least one mutation.
2. **Simulated sequence evolution:** We assign a random DNA sequence of length 50 to 1,000 nucleotides to the root of the tree, with the length chosen to reflect the plausible expected number of observable segregating sites.
3. **Model of sequence evolution:** Using the Hasegawa, Kishino, and Yano (HKY) model,^86^ we simulate sequence evolution along the tree branches to generate sequences for the descendant taxa.
4. **Multiple sequence alignment (MSA):** The evolved sequences are formatted into a multiple sequence alignment using a Python script.
5. **Cell lineage inference using MEGA-CC** ^87^: The aligned sequences are used as input for the reciprocal of the sequence length in the reconstruction of the cell lineage tree using the minimum evolution (ME) method.^88^ The ME method combines the simplicity and computational feasibility of distance methods with a clear optimality principle (minimizing total tree length), giving it a theoretical advantage over NJ (heuristic only) and practical advantages over MP (bias-prone) and ML (computationally heavy). Pairwise evolutionary distance matrices were computed using the maximum composite likelihood (MCL) method implemented in MEGA.^89^ The inference process is handled via the run_mega_me_tree() function, which includes automatic retry mechanisms to handle potential execution failures.
6. **Tree comparison and accuracy measurement:** The inferred cell lineage tree is compared to the original true tree using the Robinson-Foulds (RF) distance,^90^ a widely used metric for measuring the dissimilarity between trees. The correctness of tree inference is computed as
  (15) $$\mathrm{Correctness}=1-\frac{\mathrm{RF}\;\mathrm{Distance}}{\mathrm{Max}\;\mathrm{RF}\;\mathrm{Distance}},$$
  where a correctness value of 1.0 indicates a perfect reconstruction. In this study, our primary objective is to reconstruct cell history rather than to cluster cells by type; accordingly, we used RF distance as the principal evaluation metric. We emphasize that ‘cell type’ is a concept emergent from lineage relationships rather than an innate and static property of cells. In other words, cell differentiation represents a continuum rather than a set of discrete states, and thus distinct clusters are not generally expected.^91^
7. **Iterative simulations:** The entire process is repeated 500 times for each sequence condition (defined by sequence length, randomly generated tree topology, and evolutionary model) to obtain statistically robust results. The mean and standard deviation of correctness scores are computed.

We repeated the procedure above, varying the average branch length (expected substitutions per site) from $10^{-6}$ to $10^{0}$ (covering plausible ranges), using a simulated DNA sequence of 1,000 nucleotides.

### 2.6. Modelling scRNA-Seq–specific noises

To emulate scRNA-seq observations, we also applied a noise model to the evolved sequences at the level of individual cells. This model captures multiple sources of technical and biological noises characteristic of scRNA-seq data, including ADO,^26^ limited and heterogeneous transcript coverage,^22^ RNA editing,^92^ and sequencing errors.^93^

For each cell–gene combination, the true haploid sequence was converted into an observed consensus sequence by explicitly simulating a finite number of RNA reads. Read start positions were sampled along the transcript, and individual bases within reads were subject to stochastic errors according to empirically motivated per-base error rates. Unless otherwise stated, simulations used a mean read depth of 200 reads per gene and a read length of 75 bp, reflecting typical scRNA-seq coverage of moderately expressed transcripts.

ADO was modelled as the complete absence of a detectable signal for a given cell–gene pair with probability $p_{\mathrm{ADO}}$, representing failures in transcription, reverse transcription, or amplification. To assess the robustness of lineage reconstruction under varying levels of dropout, we performed a parameter set of $p_{\mathrm{ADO}}=0.02,\;\;0.1,\;\;0.2,\;\mathrm{and}\;\;0.5$. For the most extreme dropout condition ($p_{\mathrm{ADO}}=0.5$), gap-based site filtering was disabled, as a standard gap threshold (0.2) would otherwise eliminate nearly all sites. In all simulations, no additional per-base dropout within reads was applied (per-base  dropoutprobability=0).

Base-level substitutions were introduced independently to represent sequencing and amplification errors, with a per-base sequencing error rate of 0.001. RNA-editing–like events were also incorporated, including A→G and C→T substitutions with rates of 0.002 and 0.0005, respectively. These values are consistent with reported error and editing rates in scRNA-seq data (Table 1).

Because this noise model operates at the level of individual reads and individual cells for each gene, the overall simulation framework is computationally intensive. Each simulation replicate requires gene-by-gene sequence evolution, per-cell read simulation, extraction of segregating sites, and phylogenetic reconstruction. Consequently, exhaustive parameter sweeps over the full range of mutation loads or segregating site counts are computationally prohibitive.

To address this limitation, we performed a sparse exploration of the parameter space governing mutation burden, expressed either as the number of segregating sites or as mutations per gene. We employed a reduced somatic genome comprising 200 genes to generate scRNA-seq data, with representative parameter values spanning mutation regimes corresponding to approximately 100 to 4,000 segregating sites. This strategy enabled us to characterize both qualitative and quantitative relationships among mutation burden, segregating site availability, and tree reconstruction accuracy while maintaining computational feasibility.

Distributing mutations across independent loci increases the number of phylogenetically informative segregating sites while reducing saturation and homoplasy. This principle is well established in both theoretical phylogenetics and genome-scale empirical studies.^94–98^

Accordingly, this simulation paradigm is expected to yield higher reconstruction accuracy than previous simulations that concentrated mutations within a single locus, given the same total number of segregating sites. For comparison, we also resimulated somatic genome evolution in the absence of scRNA-seq–specific noise.

### 2.7. Simulation framework for comparing direct phylogenetic signal and genotype-mediated inference frameworks

To evaluate the performance of RTCA relative to genotype-mediated inference pipelines implemented in PhylinSic,^29^ we constructed a reproducible simulation framework that generated synthetic scRNA-seq data with realistic mutation and noise processes. The workflow was implemented in Python and executed in parallel using the ProcessPoolExecutor module.

For each replicate, a rooted phylogenetic tree representing the lineage relationships among cells was generated. The number of terminal taxa (cells) was set to 100 unless otherwise specified. Branch lengths were sampled such that the mean branch length was $10^{-3}$ substitutions per site and was further scaled by factors of 0.1, 0.25, 0.5, 0.75, and 1.0 to represent different mutation-rate regimes consistent with the extremely low mutation burden expected in normal tissues. The resulting tree served as the ground truth for evaluating lineage reconstruction accuracy.

Somatic mutations were simulated under an infinite-sites–like process along the branches of the lineage tree.^45,53^ For each simulated gene segment of length *L*, mutation events were randomly placed on tree branches with probability proportional to branch length. The total number of events was drawn from a Poisson distribution with mean proportional to the total tree length. Mutation sites were sampled without replacement to prevent multiple substitutions at the same site. Each mutation was inherited by all descendant cells of the branch on which it occurred, producing a genotype matrix across simulated cells. The full simulation consisted of multiple genes, each containing an independent sequence of length 1,000 nucleotides.

To approximate realistic scRNA-seq measurements, simulated genotypes were subjected to several sources of technical and biological noise, including ADO and sequencing errors.^26,70^ The ADO rate was systematically varied across simulation conditions (eg, 0.1 to 0.5) to evaluate the robustness of lineage inference under increasing levels of dropout.

For the RTCA pipeline, lineage reconstruction was performed using site-pattern representations derived directly from the simulated read data. Sites with sufficient coverage across cells were retained, and cell-specific consensus states were concatenated to form a pattern alignment matrix. Sites with excessive missing data were filtered using a missing-fraction threshold (max_missing_frac), ensuring that only sites with sufficient observations across cells were retained. The resulting alignment therefore contained only sites that passed the quality-control thresholds.

For the genotype-mediated inference pipeline, simulated read counts were converted into reference and alternative allele counts. These matrices were processed using the PhylinSic smoothing algorithm implemented in the smoothing.R script.^29^ PhylinSic infers surrogate genotypes for each cell at each variant site using a smoothing procedure that incorporates allele counts across neighbouring sites and cells. The resulting genotype calls were converted to nucleotide sequences and concatenated into FASTA alignments suitable for phylogenetic reconstruction.

Phylogenetic trees were reconstructed from both RTCA and PhylinSic alignments using the ME method implemented in MEGA-CC.^99^ Identical phylogenetic inference settings were applied to both methods to ensure a fair comparison.

Each simulation condition consisted of multiple independent replicates. To ensure reproducibility, replicate-specific random seeds were deterministically derived from the master seed, the ADO rate, and the replicate index.

The accuracy of inferred lineage trees was quantified using the RF distance between the inferred tree and the true simulated tree.^90^

For each replicate, the following metrics were recorded: RTCA tree correctness, PhylinSic tree correctness, the alignment length produced by each method, the number of taxa retained after filtering, and the simulation parameters (eg, the ADO rate). These results were aggregated across replicates to compute the mean and standard deviation for each simulation condition.

### 2.8. Single-cell RNA-Seq data

In this study, we used high-quality scRNA-seq data generated by Pavličev et al.^100^ available under SRA Study ID **SRP090944**. The dataset includes 2 normal placental tissue samples from genetically distinct individuals who did not share any de novo somatic mutations (Batch 1: 54 cells, SRR4371525; Batch 2: 33 cells, SRS1732319) (see Tables S1 and S2 for details). Notably, the data were sequenced at a median depth of 3.65 million reads per cell, substantially exceeding the typical depths of standard single-cell RNA-seq studies,^70^ thereby enabling more reliable variant calling.

In their original analysis, Pavličev et al. explored receptor–ligand gene expression networks to infer cell–cell communication between the semi-allogenic individuals (mother and fetus). They identified cell-type-specific expression of G-protein-coupled receptors, suggesting that ligand–receptor signalling profiles may serve as robust markers for cell type classification. Based on principal component analysis (PCA) of gene expression profiles and 300 marker genes, the authors categorized the cells into 5 distinct types: cytotrophoblast 1 (CYT1), CYT2, CYT3, extravillous trophoblast (EVT), and a putative maternal dendritic cell (DC).

### 2.9. Mapping of sequence data

From the above scRNA-seq datasets, we obtained multiple alignment data that represent single cells by aligning the homologous regions of their transcriptomes. To this end, reads were assigned designated coordinates of the reference genome. The entire data analysis pipeline is shown in Figure S2. We mapped single-cell transcriptome sequence data to the human genome (GRCh38)^101^ using the Burrows-Wheeler Aligner^102^ after removing adaptor sequences with trimmomatic.^103^ We identified variants by comparing the aligned read sequences with the reference genome using Samtools.^102^ In this study, we used only SNVs, excluding all detected indel events. The breadth of coverage can vary across transcriptome data among cells, so there were some missing data in the alignments. In healthy tissues, however, mutation rates are typically uniform across expressed exonic regions, with hotspots observed in actively transcribed genes.^12^ Assuming uniformity in our data, we excluded sites with more than 50% missing data across cells.

### 2.10. Phylogenetic analysis of single cells

We concatenated all detected variant sites to generate sequence alignments and reconstructed cell lineage trees using the ME method^88^ implemented in MEGA X.^87^ The analysis was performed with uniform rates among sites, pairwise deletion for gaps and missing data, and an ME search level of 1. Pairwise distances were estimated using the MCL method,^89^ topology searches were conducted using the close-neighbour interchange algorithm,^88^ and initial trees were generated using the neighbour-joining (NJ) method.^104^ The complete set of parameters is provided in the Supplementary material. We also computed the confidence probability that each internal branch length was greater than zero using 100 bootstrap replications, and the resulting trees were output in Newick format.^105^

### 2.11. A simulation comparison with the real data

To associate our simulation analyses with the empirical scRNA-seq dataset, we used 3 empirically observed quantities as reference values: the Batch 1 sample size of 53 cells, the estimated average branch length of 0.0217 nucleotide substitutions per site, and the observed number of variant sites (1,335).

First, we evaluated how phylogenetic reconstruction accuracy depends on average branch length. Simulated datasets were generated across a range of mutation densities, and phylogenetic trees were reconstructed for each condition. Mean correctness and standard deviation were calculated across replicate simulations as described above. The empirical average branch length of 0.0217, estimated from the Batch 1 analysis of 53 cells, was then projected onto the simulation-derived curve to estimate the expected reconstruction accuracy under the empirical branch-length regime.

Second, we evaluated reconstruction accuracy as a function of the number of observed variant sites. Simulations were performed across increasing numbers of available variant sites, and correctness was summarized across replicates. The empirical value of 1,335 observed variant sites was overlaid onto the simulation curve to estimate the expected accuracy corresponding to the variant-site availability observed in the real dataset.

Third, to assess the robustness of reconstruction accuracy to scRNA-seq–specific noise, we repeated the variant-site simulations under different ADO conditions: no ADO, ADO = 0.02, ADO = 0.1, and ADO = 0.2. For each ADO condition, mean correctness and standard deviation were calculated across replicates as a function of the observed variant-site count. The empirical value of 1,335 variant sites was then used as a common reference point to interpolate the expected correctness under each dropout condition.

Finally, the interpolated correctness values at 1,335 variant sites were summarized across ADO conditions. This analysis provided an empirical-data–anchored estimate of the extent to which reconstruction accuracy would be expected to decline with increasing allelic dropout while keeping the observed variant-site availability fixed. Overall, these simulations were designed to determine whether the empirical dataset lies within a parameter regime in which accurate lineage reconstruction is theoretically expected.

### 2.12. Gene expression reanalysis

For linear dimensionality reduction, we conducted PCA on the expression patterns using R (version 3.6.2),^106^ followed by applying t-SNE^47^ and UMAP^48^ for nonlinear dimensionality reduction. The Louvain method was then used for clustering.^107^

### 2.13. The pseudotime analysis

We conducted a pseudotime analysis using monocle3 (version 1.0.0)^108–110^ on R (version 4.1.2).^106^ Pseudotime analysis estimates the temporal dimension of static single-cell transcriptome data and infers individual gene expression dynamics along with cellular changes. We reduced the data dimension to 26 using PCA. To visualize the results, we used *Mathematica*.^111^

#### 2.13.1. Comparative analysis of mutation patterns and gene expression profiles

**Fig. 2.**
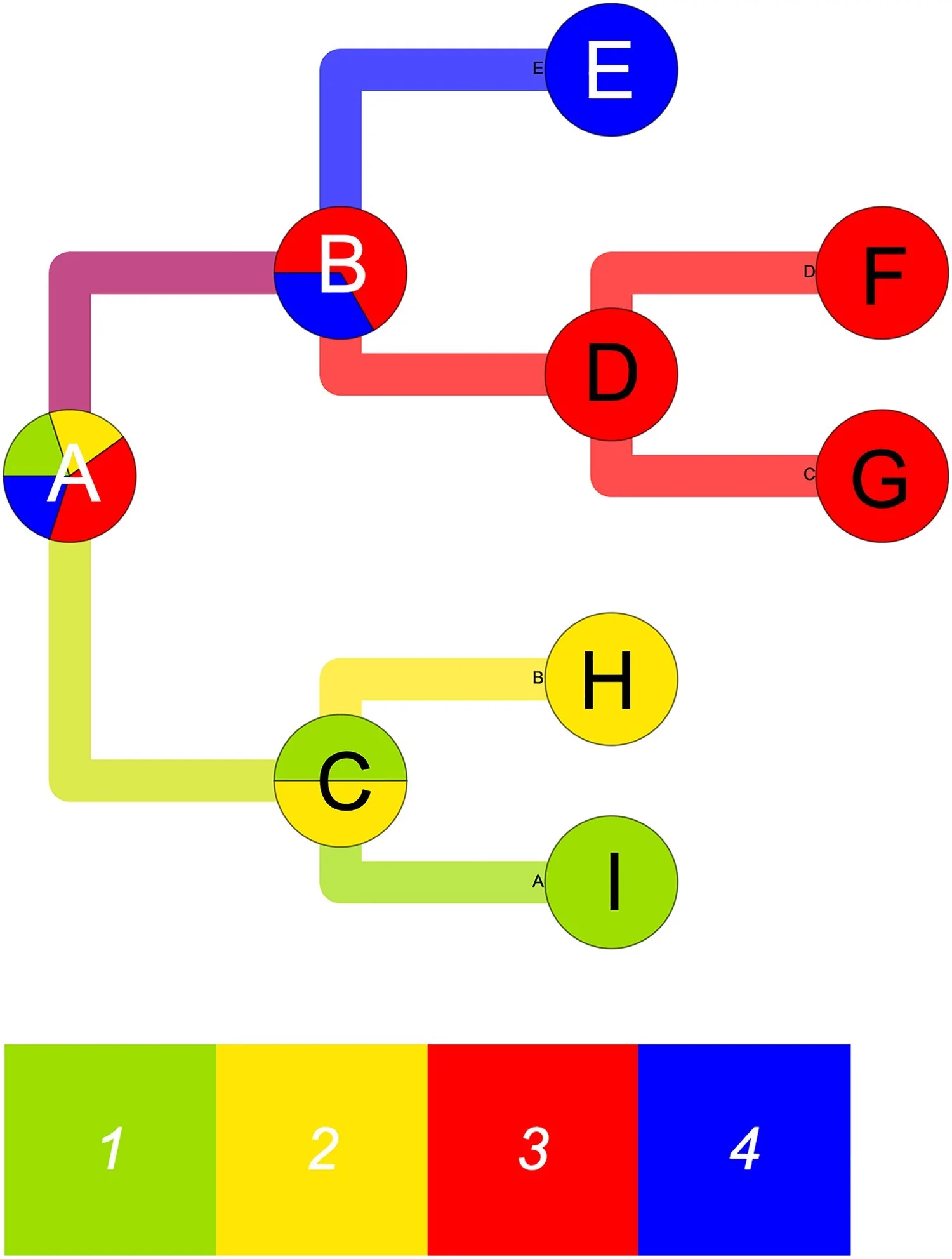
Visualization of the degree of monophyleticity (DoM) at each node, based on the proportion of clusters in its subtree. The gene expression profile (represented by a cluster table) is mapped onto the tree topology. When the clustering is consistent with the tree structure, subtrees appear monophyletic with respect to the clustering. Otherwise, para- or polyphyletic subtrees may arise. The AssignCluster2Cell code calculates the proportion of cells assigned to clusters within each subtree, enabling evaluation of the DoM for monophyly. A: an inferred ancestral cell. B, C, and D: inferred derived cells. E, F, G, H, and I: observed differentiated cells. Clusters: 1 (I: Green), 2 (H: Yellow), 3 (F and G: Red), and 4 (E: Blue) correspond to the cluster table. Edge colours are blended based on the colours of the connecting nodes recursively representing cell clusters.

We mapped the clustered cells to the cell lineage trees, which were reconstructed using cell genotypes. To this end, we developed a *Mathematica*^111^ code, *AssignCluster2Cell*, using 2 packages: *Phylogenetics for Mathematica*^112^ and *Phylogenetics*.^113^  *AssignCluster2Cell* reads a Newick^105^ formatted tree file and a cluster table of a gene expression profile, and maps cluster ID to the tree topology, evaluating degrees of monophyleticity^114^ (DoMs)—an extended version of the traditional degree of monophyly^115,116^—at each node, is defined as the proportion of cluster members contained within its subtree (Fig. 2). The DoM is particularly useful for visualizing RTCA trees.

We mapped the clustered cells based on gene expression profiles to the tree to evaluate the DoMs. The pie charts in Fig. 2 show the DoM at each node; for example, if the pie chart at a node has only one slice, the subtree is completely monophyletic (eg, Node D in Fig. 2).

Theoretically, the root of a cell lineage tree (Node A in Fig. 2) represents the zygotic cell. However, in this study, the root of the estimated tree represented a progenitor cell of the observed cells. The zygotic cell is expected to lie somewhere between the root and the reference genome.

### 2.14. Quantifying alignment–tree inconsistency under the infinite sites model

We analyzed multiple sequence alignments constructed from putative somatic SNVs. Each alignment column corresponded to a genomic site and was encoded as ancestral (0), derived (1), or missing (NA).^51,52^ To reduce trivial or uninformative cases, we excluded alignment columns that satisfied any of the following criteria: (i) all entries identical (all 0 or all 1), (ii) fewer than one observed derived allele or fewer than one observed ancestral allele, or (iii) excessive missingness (>20% of cells). Only biallelic sites were considered for simplicity.^45,76^

Under the infinite sites model (ISM),^53^ each mutation occurs at most once on the phylogenetic tree, and all descendant cells of the branch on which the mutation arises inherit the derived allele.^117^ Consequently, the set of cells carrying the derived allele at a site must form a single clade in the inferred tree.

For each alignment column *s*, we define the set of cells carrying the derived allele,


$$S_{s}=\{i|A_{is}=1\},$$

where $A_{is}$ denotes the allele state of cell *i* at site *s*.^45,76^ We identify the lowest common ancestor (LCA) of the leaves in $S_{s}$ and denote by $D_{s}$ the set of all descendant leaves of this LCA.^118^ A site is considered strictly consistent with the tree if and only if $S_{s}=D_{s}$.

Because scRNA-seq data are subject to substantial ADO and missing observations,^26,70,119^ we adopt a missing data–tolerant definition as the primary metric. Under this criterion, a site is considered consistent if all observed derived alleles are contained within a single clade and no observed ancestral alleles are present in that clade. Formally, let $D_{s}$ be the descendant set of the LCA of observed derived alleles. Then, a site is deemed consistent if:

- no observed derived alleles are found outside $D_{s}$, and
- no observed ancestral alleles are found within $D_{s}$;

missing values are allowed in either set ($D_{s}$ or its complement).^117^

In sensitivity analyses, we further consider a relaxed criterion allowing up to *k* observed ancestral alleles within $D_{s}$ (with k=1 or 2), reflecting ADO or rare back-mutation–like events.^118^

For a given tree–alignment pair, we quantify the empirical inconsistency rate^117^ as


$$\hat{\epsilon }=\frac{N_{\mathrm{inconsistent}}}{N_{\mathrm{analyzed}}},$$

where $N_{\mathrm{inconsistent}}$ is the number of alignment columns that violate the ISM-based consistency criterion, and $N_{\mathrm{analyzed}}$ is the total number of alignment columns passing the preprocessing filters.^51,76,118^ This quantity represents a tree-aware, data-driven estimate of the effective error rate in the real data, incorporating contributions from sequencing error, ADO, alignment artefacts, and model violations.^26^

Our analyses were implemented in Python. Phylogenetic trees were parsed in Newick format, and descendant leaf sets for all internal nodes were precomputed. For each alignment column, consistency was evaluated by computing the LCA of derived-allele leaves and testing the corresponding descendant set against the observed allele pattern. This approach scales linearly with the number of sites and allows efficient evaluation of large alignments.

To aid interpretation, we considered a simple probabilistic model in which site-level inconsistency arises from 2 sources: (i) true biological homoplasy due to finite-sites effects^120^ and (ii) observation error, including ADO and false-positive variant calls.^26^ Under a Poisson mutation process on the inferred tree, the probability of 2 or more mutations occurring at the same site provides a lower bound on the expected inconsistency rate due to genuine departures from the ISM. Observation-error–induced inconsistencies were modelled as a function of clade size, dropout probability, and false-positive rate.^26^ Although these parameters were not explicitly fitted in the main analysis, this framework provided an interpretable link between the empirical inconsistency rate and underlying biological and technical error processes.^45^

### 2.15. Formalization of the posterior distributions under genotype-mediated inference and direct phylogenetic signal frameworks

To make the distinction between genotype-mediated inference framework and direct phylogenetic signal framework lineage trees explicit, we formalize the posterior distributions of genotype-mediated inference framework and direct phylogenetic signal framework lineage trees using the following notation:

- Cells: i=1,…,N.
- Sites/variants: s=1,…,S.
- Tree (topology + branch lengths): *T*.
- Reads (raw sequencing data): $R=\{r_{i,k}\}$, where $r_{i,k}$ denotes the *k* -th read originating from cell *i*; in droplet-based scRNA-seq data, the cell index *i* is defined by a cell-specific barcode.
- Barcode/cell-assignment mechanism: *B* (including barcodes and whitelisting; in one-BAM-per-cell designs, *B* is trivial or degenerate).
- Latent per-cell genotype or mutation state: $G=\{g_{i,s}\}$, eg, $g_{i,s}\in \{0,1\}$ for absence/presence (or multinomial states for mitochondrial heteroplasmy^32^).
- Observed cell-associated site patterns: $Y=\{y_{i,s}\}$, where $y_{i,s}\in \{0,1,?\}$ (or {A,C,G,T,?}).
- Nuisance parameters capturing observation noise (dropout, sequencing error, allelic imbalance, etc.): *θ*.

#### 2.15.1. Genotype-mediated inference framework pipelines

The defining structural feature of genotype-mediated inference frameworks is the introduction of an explicit latent genotype (or mutation-state) layer *G*. In practice, inference is typically separated into 2 stages: (i) inference of G from reads (denoising/smoothing) and (ii) inference of T from *G* (phylogenetic reconstruction).

The posterior distribution over trees given reads can be written as


(16) $$P(T|R,B)\propto \;P(T)\int \underset{G}{\sum }\;P(R|G,B,\theta )\;P(G|T)\;P(\theta )\;d\theta .$$

Here, P(R∣G,B,θ) is a read-level emission model conditional on cell definition and genotype; this is where barcode-defined cell identity plays a role, even if barcodes do not explicitly appear in downstream steps. P(G∣T) denotes the phylogenetic prior or likelihood governing genotype evolution along the tree *T* (eg, infinite sites, finite sites, or mitochondrial mixture/heteroplasmy models).

In practice, our implementations approximate Eq. (16) by first estimating genotypes,


(17) $$\hat{G}=\arg\underset{G}{\max}\;P(G|R,B)\;\mathrm{or}\hat{\;P}(g_{i,s}=1|R,B),$$

and then inferring a tree conditional on these estimates,


(18) $$P(T|R,B)\approx P(T|\hat{G})\;\propto \;P(T)\;P(\hat{G}|T).\;$$

In this framework, missing data^22,26^ are problematic because they render P(G∣R,B) weak or unstable, often necessitating heavy smoothing or filtering.

#### 2.15.2. RTCA: a direct phylogenetic signal framework formulation

In contrast to the genotype-mediated inference framework, RTCA operates directly on observed site patterns *Y* (cell-associated sequences) with explicit modelling of missingness.^22,26^ The posterior distribution is


(19) P(T∣Y)∝P(T)∫P(Y∣T,ϕ)P(ϕ)dϕ,

where *ϕ* parameterizes the pattern-level observation model (including sequencing error and missingness), without introducing a per-cell latent genotype layer that must be “completed” prior to tree inference.

A standard decomposition of the likelihood is


(20) $$P(Y|T,\phi )=\overset{S}{\underset{s=1}{\prod }}\;\underset{a_{s}}{\sum }\;P(a_{s}\;|\;T)\overset{N}{\underset{i=1}{\prod }}\;P(y_{i,s}\;|\;a_{i,s},\phi ),$$

where $a_{s}$ denotes the latent ancestral state at site *s*. These $a_{s}^{\prime }s$ are the usual phylogenetic nuisance variables that are integrated out within the likelihood and should not be interpreted as per-cell genotypes.

Missing data are handled explicitly, for example, by defining


(21) $$P(y_{i,s}=?\;|\;a_{i,s},\phi )=\rho_{i,s},$$

or by using a more detailed, model-based missingness formulation.

RTCA therefore integrates over standard phylogenetic hidden states while treating the observed site-pattern matrix *Y* itself as the data object. It does not require inferring a “true” genotype vector for each cell as a separate inferential step.

#### 2.15.3. Conceptual contrast

In genotype-mediated inference framework pipelines, the tree *T* is inferred *through* a latent genotype layer:


(R,B,θ)→denoiseG→T.

In RTCA, by contrast, *T* is inferred directly from the pattern likelihood:


Y→(phylogeny+missingness)→T.

Even when *Y* is computed from *R*, the 2 approaches differ fundamentally in what is treated as the inferential target. Formally,


(22) $$P(T|R,B)=\underset{Y}{\sum }\;P(T|Y)\;P(Y|R,B).$$

RTCA corresponds to directly modelling P(T∣Y) by conditioning on *Y*, whereas genotype-mediated inference frameworks first estimate P(G∣R,B) and then infer P(T∣G).

In low-mutation or sparse-coverage regimes, P(G∣R,B) is often weak for most cells and sites, as many $g_{i,s}$ are nearly unidentifiable.^121,122^ Under Eqs. (16)–(18), this leads to heavy smoothing or genotype matrices dominated by missingness. In contrast, RTCA-style likelihoods (Eqs. (19)–(21)) can still accumulate phylogenetic signal by scoring the compatibility of observed site patterns under the tree while treating missingness as an explicit outcome of the observation process.

## 3. Results

### 3.1. Performance evaluation of the RTCA method via simulation

To assess the performance of the RTCA method, we conducted simulations by varying the average branch lengths and the number of variant sites in model trees. Tree reconstruction accuracy was quantified using similarity, defined by the correctness metric in Eq. (15), between the true and inferred trees.

### 3.2. Effect of branch length

**Fig. 3.**
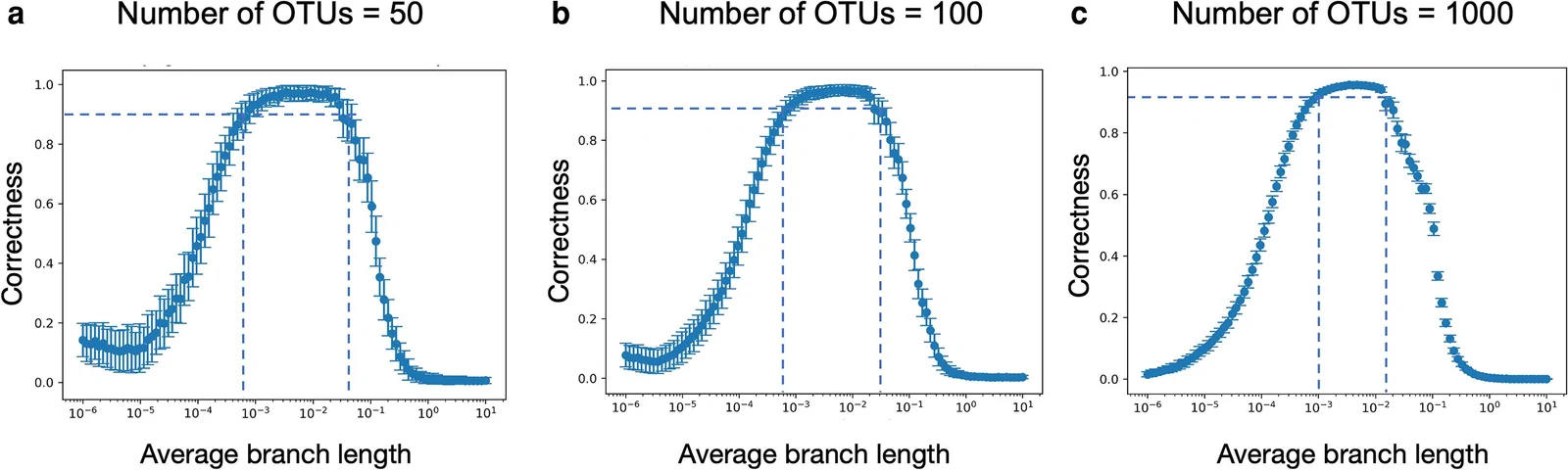
Simulation-based evaluation of cell lineage tree reconstruction accuracy across varying average branch lengths. Phylogenetic reconstruction accuracy (correctness, defined as 1 minus the Robinson–Foulds distance) is shown as a function of the average branch length of randomly generated model trees, plotted on a logarithmic scale. Results are shown for trees with different numbers of OTUs: (a) 50 OTUs (first parameter setting), (b) 100 OTUs (second parameter setting; see Methods), and (c) 1,000 OTUs. Each point represents the mean correctness across 500 independent simulation replicates, and error bars denote the standard deviation. Across all tree sizes, reconstruction accuracy exhibits a characteristic “sweet spot” at intermediate branch lengths, where phylogenetic signal is maximized. Very short branches provide insufficient numbers of segregating sites, resulting in low accuracy, whereas excessively long branches lead to mutational saturation and homoplasy, again reducing reconstruction performance. The horizontal dashed line marks a reference accuracy level (0.9), and the vertical dashed lines indicate the empirically determined optimal branch-length range that achieves near-maximal accuracy. As the number of OTUs increases, this optimal window shifts and becomes narrower, reflecting the increased difficulty of resolving larger and more complex trees.

Figure 3 illustrates how average branch length influences phylogenetic reconstruction accuracy across different tree sizes. In all cases, reconstruction performance exhibited a characteristic “**sweet spot”** at intermediate branch lengths, where accuracy was maximized. When branches were too short, too few mutations accumulated along lineages to generate sufficient phylogenetic signal, resulting in poor reconstruction accuracy. In contrast, excessively long branches led to mutational saturation and increased homoplasy, which again reduced inference performance.

This optimal branch-length regime was consistently observed across trees with different numbers of cells (OTUs), including 50, 100, and 1,000 taxa (Fig. 3a–c). However, as tree size increased, the effective window of branch lengths yielding high accuracy became progressively narrower and shifted slightly, reflecting the increased difficulty of resolving larger and more complex trees. Overall, these simulation results are fully consistent with theoretical expectations regarding the trade-off between signal accumulation and saturation as a function of branch length (Fig. 1).

### 3.3. Effect of variant site number

**Fig. 4.**
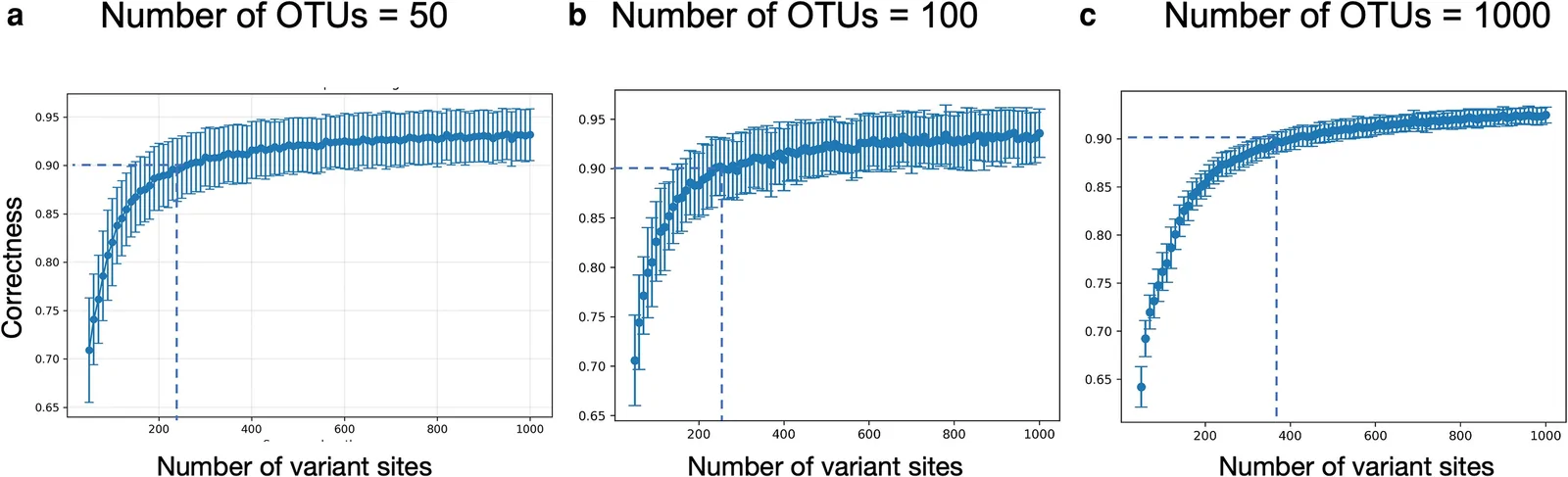
Simulation-based evaluation of cell lineage tree reconstruction accuracy as a function of variant site availability and tree size. Mean phylogenetic reconstruction accuracy (“correctness”; mean ± s.d.) is plotted against the number of variant (segregating) sites in the alignment for randomly generated binary trees with different numbers of OTUs (cells): (a) 50, (b) 100, and (c) 1,000. Points indicate the average correctness across 500 replicate simulations per condition, and error bars denote the standard deviation. In all tree sizes, correctness increased rapidly with increasing numbers of variant sites and then plateaued, indicating diminishing returns once sufficient phylogenetic signal accumulated. The horizontal dashed line marks a target accuracy level (0.90), and the corresponding vertical dashed line indicates the approximate number of variant sites required to reach this threshold: ∼250 sites for 50 OTUs, ∼270 sites for 100 OTUs, and ∼380 to 400 sites for 1,000 OTUs. These results show that the number of variant sites required for reliable reconstruction increases with tree size, consistent with greater topological complexity in larger trees.

We also evaluated the impact of sequence length on reconstruction correctness (Fig. 4). As expected, increasing the number of variant sites improved reconstruction accuracy. For trees with 100 OTUs, approximately 300 variant sites were sufficient to achieve 90% accuracy (Fig. 4b), while larger trees with 1,000 OTUs required around 400 variant sites to reach comparable levels of accuracy (Fig. 4c).

The above findings indicate that both branch length and sequence length should be at appropriate levels to ensure reliable cell lineage reconstruction from somatic variants.

Importantly, as described below, the variant data obtained from the placenta scRNA-seq dataset satisfy both conditions.

### 3.4. Effect of scRNA-Seq–specific noise on phylogenetic reconstruction accuracy

**Fig. 5.**
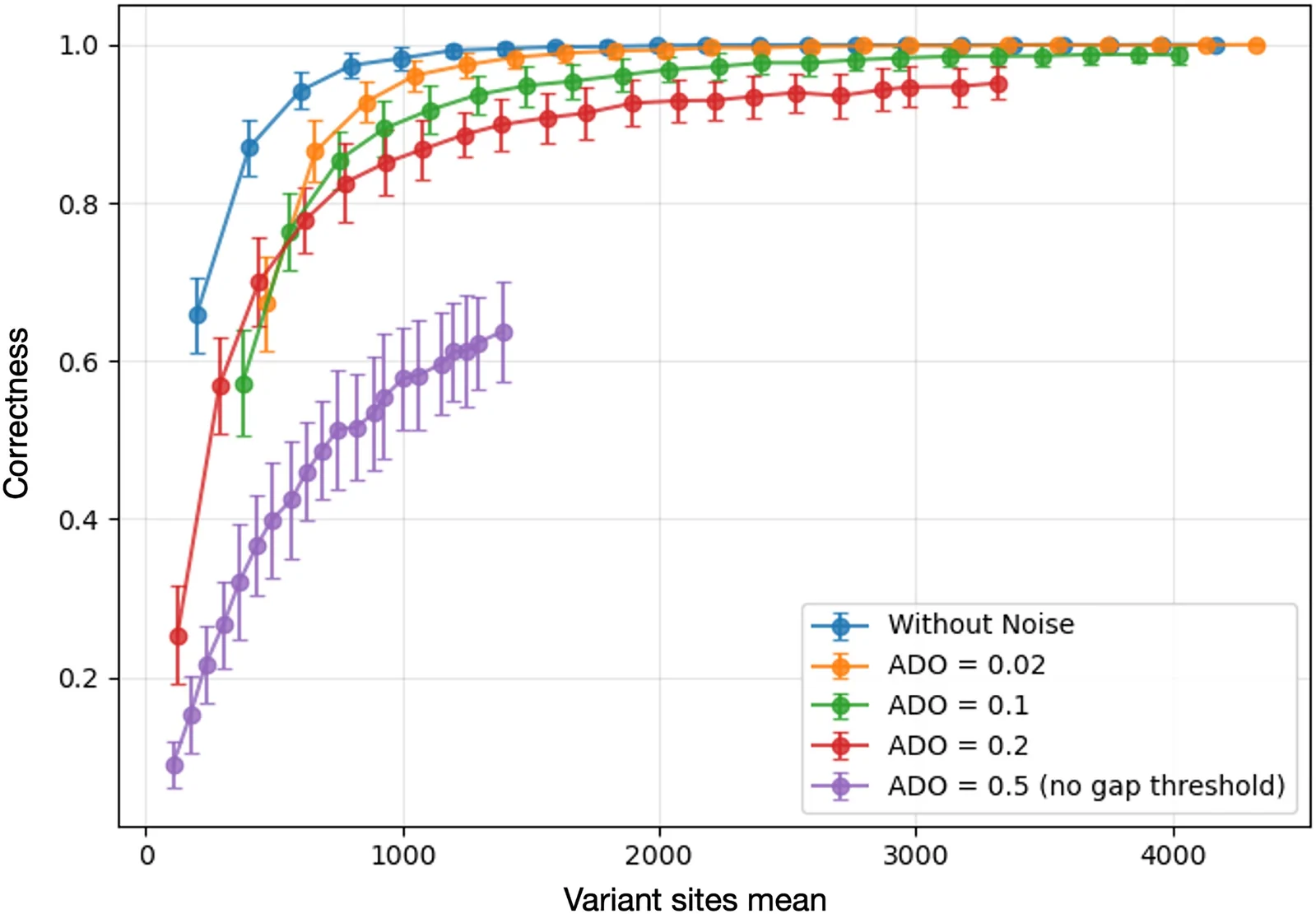
Effect of scRNA-Seq–specific noise on phylogenetic reconstruction accuracy. Phylogenetic reconstruction accuracy, quantified as the mean correctness score (± s.d.; *n* = 100), is shown as a function of the mean number of observed segregating sites across concatenated loci. Results are shown for simulations without scRNA-Seq–specific noise and with increasing levels of ADO (ADO=0.02,0.1,0.2,and0.5). In the absence of noise, accuracy increases rapidly and approaches saturation with approximately 800 to 1,000 segregating sites. Incorporation of scRNA-Seq–specific noise shifts the accuracy curves rightward, indicating that a larger number of informative sites is required to achieve comparable performance. Under a higher ADO, accuracy improves more gradually and exhibits increased variability but continues to increase monotonically with additional segregating sites. The spacing of data points along the horizontal axis is uneven because the number of segregating sites was not directly controlled, but instead emerged stochastically from simulations parameterized by discrete mutation-per-gene values under different noise conditions. As a result, identical mutation burdens can yield different numbers of observable segregating sites depending on the ADO rate and filtering thresholds. These results demonstrate that scRNA-Seq–specific noise primarily reduces the efficiency of phylogenetic signal recovery rather than imposing a strict upper limit on achievable accuracy. For the simulation with ADO=0.5, no gap threshold was applied because any gap-based filtering would have removed all variant sites under this level of allelic dropout.

Figure 5 summarizes the relationship between the availability of segregating sites and phylogenetic reconstruction accuracy under increasing levels of scRNA-seq–specific noise. Accuracy is shown as the mean correctness score (± s.d.; *n* = 100) plotted against the mean number of observed variant sites across concatenated loci.

In the absence of scRNA-seq noise, reconstruction accuracy increased rapidly with the number of segregating sites, reaching near-perfect performance with approximately 800 to 1,000 variant sites. Beyond this range, additional segregating sites provided diminishing returns, and accuracy plateaued close to the theoretical maximum. This behaviour is consistent with expectations from phylogenetic theory, in which a modest number of informative sites is sufficient to resolve tree topology when noise and homoplasy are minimal.

Note that the discrepancy between these results (Fig. 5) and those of the previous simulation (Fig. 4) arises from differences in the simulation paradigm. As described in Materials and Methods, the present simulations distribute mutations across independent loci—a regime that naturally reflects transcriptional organization—and this distribution increases the number of phylogenetically informative segregating sites while reducing saturation and homoplasy.^94–98^ Consequently, this framework yields higher tree-reconstruction accuracy than the previous simulation under comparable mutation burdens.

Incorporation of scRNA-seq–specific noise resulted in a systematic rightward shift of the accuracy curve. With low ADO (ADO = 0.02), higher numbers of segregating sites were required to achieve the same level of accuracy observed under noiseless conditions. Nevertheless, accuracy still approached saturation at high mutation loads, indicating that increased site availability can partially compensate for transcriptomic noise.

Under more severe ADO (ADO=0.1 and 0.2), accuracy increased more gradually and saturated at slightly lower levels. In this regime, several hundred additional segregating sites were required to achieve high reconstruction accuracy, and variability across replicates was elevated, reflecting increased stochasticity in the observed data. Despite this, reconstruction performance continued to improve monotonically with increasing availability of variant sites, indicating that phylogenetic signals are preserved even under substantial scRNA-seq–specific noise.

Under extremely severe ADO (ADO = 0.5), the standard gap threshold (0.2; see Materials and Methods) filtered out all variant sites. Therefore, the gap threshold was removed in this condition. Biologically, this scenario corresponds to extremely low transcript expression levels, under which extensive ADO is expected.^70^

The spacing of data points along the horizontal axis is uneven because the number of segregating sites was not treated as an independent variable, but instead emerged stochastically from simulations parameterized by discrete mutation-per-gene values under different noise conditions. As a result, identical mutation burdens can yield different numbers of observable segregating sites depending on the ADO rate and filtering thresholds.

Collectively, these results demonstrate that scRNA-seq–specific noise primarily affects the efficiency with which phylogenetic signals are recovered, rather than imposing a strict upper bound on achievable accuracy. Importantly, they show that reliable tree reconstruction can be achieved with relatively sparse mutation burdens, even in the presence of realistic levels of ADO. This finding supports the feasibility of lineage reconstruction from scRNA-seq data in normal tissues, where somatic mutations are expected to be rare.

### 3.5. Direct phylogenetic signal and genotype-mediated inference frameworks

The simulation results are summarized in Table 3. Across all mutation-rate regimes, lineage reconstruction accuracy declined as the ADO rate increased. For both RTCA and PhylinSic, tree correctness decreased progressively from ADO = 0.1 to ADO = 0.5, reflecting the loss of informative sites caused by dropout.^26,70^ This trend was observed consistently across all branch-length scaling factors (F).

**Table 3.** Performance comparison of direct phylogenetic signal framework (RTCA) and genotype-mediated inference framework (PhylinSic) approaches under increasing allelic dropout rates across different mutation-rate regimes.

F	ADO	RTCA		PhylinSic		RTCA Alignment length		PhylinSic Alignment length	
		Mean	Std	Mean	Std	Mean	Std	Mean	Std
1.00	**0**.**1**	0.923	0.025	0.946	0.019	936.08	32.799	936.14	32.812
	**0**.**2**	0.851	0.032	0.870	0.033	887.88	25.205	897.32	25.173
	**0**.**3**	0.758	0.050	0.709	0.046	822.40	34.370	906.86	39.882
	**0**.**4**	0.649	0.053	0.311	0.080	749.86	32.074	971.14	35.974
	**0**.**5**	0.364	0.058	0.079	0.028	378.48	22.266	982.62	40.689
0.75	**0**.**1**	0.883	0.029	0.909	0.028	705.04	27.531	705.16	27.400
	**0**.**2**	0.805	0.044	0.824	0.038	659.28	26.685	666.14	26.812
	**0**.**3**	0.691	0.053	0.607	0.058	618.32	29.496	678.58	31.360
	**0**.**4**	0.572	0.056	0.227	0.059	561.06	25.811	725.96	32.561
	**0**.**5**	0.291	0.065	0.049	0.024	288.38	20.439	744.36	31.972
**0**.**50**	**0**.**1**	0.819	0.037	0.839	0.043	473.70	24.878	473.78	24.866
	**0**.**2**	0.714	0.049	0.737	0.056	439.84	22.085	445.08	21.807
	**0**.**3**	0.578	0.068	0.496	0.072	412.38	23.070	453.16	25.755
	**0**.**4**	0.439	0.062	0.141	0.036	370.30	23.451	481.20	27.969
	**0**.**5**	0.209	0.058	0.035	0.020	191.92	12.977	491.34	19.964
0.25	**0**.**1**	0.664	0.069	0.666	0.057	236.5	15.451	236.7	15.514
	**0**.**2**	0.532	0.055	0.498	0.058	220.6	15.904	222.9	16.502
	**0**.**3**	0.384	0.058	0.249	0.063	201.5	18.686	224.3	20.106
	**0**.**4**	0.273	0.085	0.075	0.025	187.9	15.680	243.7	20.287
	**0**.**5**	0.120	0.038	0.023	0.016	99.8	13.079	246.9	17.527
0.10	**0**.**1**	0.352	0.056	0.356	0.060	94.16	9.565	94.16	9.565
	**0**.**2**	0.257	0.054	0.245	0.065	89.86	9.737	91.32	9.738
	**0**.**3**	0.195	0.055	0.111	0.043	80.06	8.966	89.32	8.599
	**0**.**4**	0.142	0.036	0.026	0.014	78.00	9.693	99.68	10.522
	**0**.**5**	0.063	0.030	0.008	0.010	38.92	6.477	98.40	9.357

Mean and standard deviation of lineage reconstruction accuracy (tree correctness) and alignment length (number of aligned nucleotide sites) across replicate simulations. Branch lengths were sampled such that the mean branch length was $10^{-3}$ scaled substitutions per site and was further scaled by factors (F) of 0.1, 0.25, 0.5, 0.75, and 1.0 to represent different mutation-rate regimes consistent with the extremely low mutation burden expected in normal tissues. Allelic dropout (ADO) was varied from 0.1 to 0.5 to evaluate the robustness of lineage inference under increasing levels of scRNA-seq–specific noise. Notably, RTCA maintains substantially higher reconstruction accuracy than PhylinSic when the mutation rate is low (small F), where the number of informative sites becomes extremely limited.

**Note:** Values represent the mean and standard deviation across replicate simulations. Alignment length indicates the number of sites retained after quality control filtering in each pipeline. RTCA and PhylinSic alignments were generated independently following their respective filtering procedures; therefore, the number of retained sites may differ between them.

A clear difference between the 2 methods emerged under low mutation-rate conditions. When the branch-length scaling factor was small (eg, F=0.1 or 0.25), the number of segregating sites retained after filtering was substantially reduced. Under these sparse-mutation conditions, RTCA maintained substantially higher reconstruction accuracy than PhylinSic, particularly at moderate to high ADO levels. For example, at F=0.1 and ADO = 0.4 to 0.5, PhylinSic accuracy dropped to near zero, whereas RTCA retained measurable reconstruction accuracy. A similar pattern was observed for F=0.25, where the decline in accuracy was considerably steeper for PhylinSic than for RTCA.

In contrast, when the mutation rate was relatively high (F=0.75–1.0), both methods achieved comparable reconstruction accuracy at low ADO levels, indicating that abundant informative sites mitigate differences between the pipelines. However, as ADO increased, the performance of PhylinSic deteriorated more rapidly than that of RTCA.

These results indicate that the advantage of RTCA becomes most pronounced in regimes where the mutation burden is extremely low, a condition expected in normal tissues.^12,123^ Under such circumstances, the direct phylogenetic signal framework formulation of RTCA appears more robust to sparse mutation signals and dropout-induced missing data than genotype-mediated inference framework approaches such as PhylinSic.^29^

### 3.6. Detection of somatic variants in the single-cell transcriptome data

In total, 1,965,629 and 830,905 variant sites were detected in Batch1 and Batch2 data, respectively, during the first screening (Figure S3). Among these, 89 multiallelic sites across all the cells were found in 30 Batch1 cells. Overall, we obtained 1,335 variant sites that were multiallelic or biallelic at least in 43.2 single cells on average (80% of the 54 single cells) in Batch1. Similarly, in Batch2, 31 sites were multiallelic in all 33 single cells. Overall, 574 sites were multiallelic or biallelic at least in 26.4 single cells on average (80% of the 33 single cells). This means that we adopted a strategy that tolerates up to 20% gaps at each site arising from ADO; that is, we effectively focused on relatively highly expressed transcripts across cells. All data met the expected read quality threshold of Q30 and exhibited high RNA integrity (RIN > 9), with an average coverage of at least 10× across expressed genes. These results are in good agreement with the theoretical expectations. Here, we selected the ‘minor allele’ (the less frequent nucleotide variant at a site when aggregating among single cells)^124^ as a derived variant, assuming that the minor alleles were newly derived nucleotides in a cell population.

### 3.7. Phylogenetic analysis of placental single-cell data

**Fig. 6.**
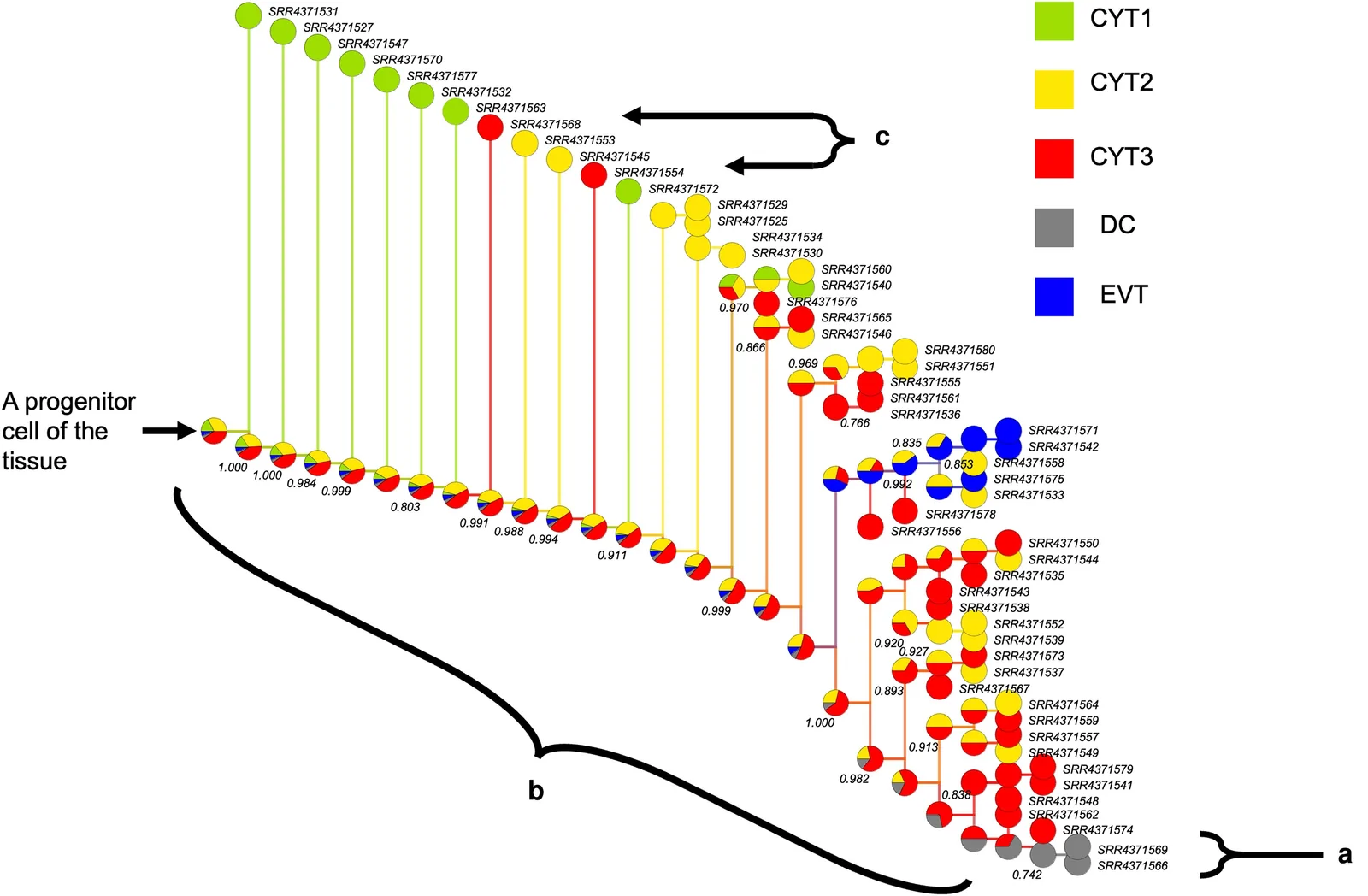
Mapping of the gene expression profile to a cell lineage tree (SRP090944 data; Batch1, 54 cells). The gene expression profile (as clustered cells) is mapped to the tree topology: External nodes represent observed cells, and internal nodes represent inferred cells, which are colour-coded according to the gene expression patterns with pie charts. (a) Placenta cells claimed as the maternal DC^100^; (b) a putative stem cell self-renewal phase; and (c) putatively erroneous locations of single cells (SRR4371568 and SRR4371554). Numbers on edges represent the confidence probability that the internal branch length is greater than zero according to 500 bootstrap resamplings. Branch colours were assigned according to the blended cell type colours within each subtree (see Fig. 2).

**Fig. 7.**
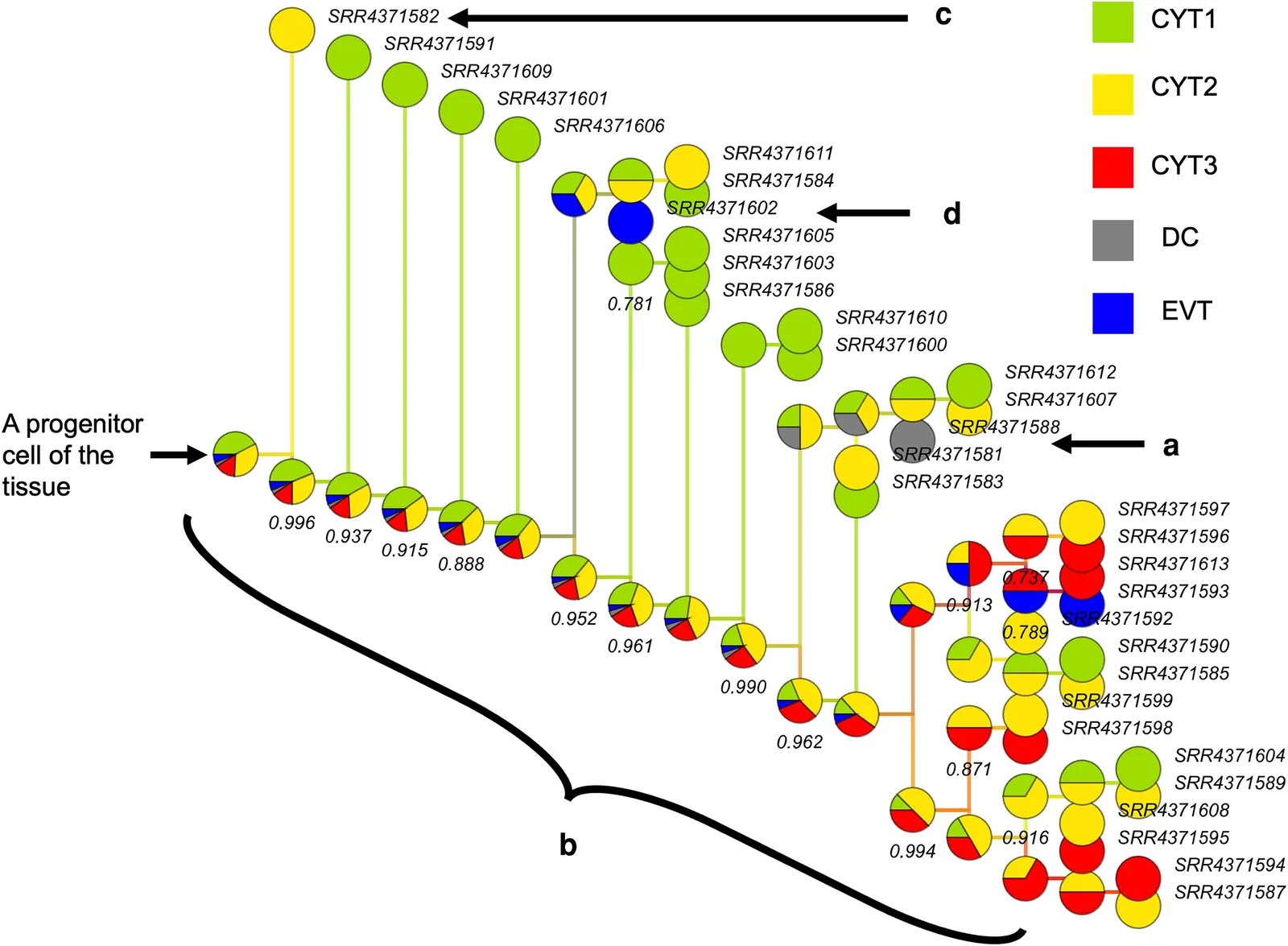
Mapping of gene expression profiles onto an inferred cell lineage tree topology (SRP090944 dataset; batch 2, 33 cells). Panels (c) and (d) indicate putatively erroneous placements of single cells SRR4371582 and SRR4371602, respectively. Other annotations follow the conventions in Fig. 6.

Using the variant call data described above, we reconstructed the phylogenetic trees of single cells from 2 different individual placental tissue samples (Figs. 6 and 7). Each leaf—an OTU—corresponds to a cell from a tissue sample. While the trees are unrooted, we assigned roots by using the human reference genome as the outgroup. The reference human genome itself is omitted from the figures for clarity.

A model of the differentiation process from villous cytotrophoblasts (vCTB) to EVTs has been proposed by Knöfler et al.^125^ in which EVT are differentiated from vCTB via cell column trophoblasts (Figure S1). Note that the terms “intravillous cytotrophoblast (CYT)” and “vCTB” are often used interchangeably, as they refer to the same cell population found within the chorionic villi of the placenta.^126^

Pavličev et al. used PCA analysis and the expression patterns of marker genes to classify the single cells into 5 clusters, corresponding to 5 cell types: intravillous cytotrophoblasts (CYT1, CYT2, and CYT3), EVT, and the putatively maternal DCs. We mapped the cell types assigned by Pavličev et al. onto the tree reconstructed by our variant calls. As shown in Fig. 6b, all 9 CYT1 cells are located on the ‘trunk of the tree’, which is considered to represent the progenitor cells of CYT2, CYT3, and EVT.

Notably, the differentiation of CYT2 and CYT3 suggests that intravillous cytotrophoblasts constitute a heterogeneous group, with differentiation occurring across a continuum of states rather than through stepwise phase transitions. The expression profiles of these cells suggest that differentiation proceeds through dynamic gene expression in varying sequences, rather than a uniform progression towards a terminal expression profile.^100^

Meanwhile, we identified a small number of apparent discrepancies in the above patterns, as shown in Fig. 6c. Figure S4 examines the relationship between coverage percentage and the cells classified as discrepant. Although such discrepancies are often attributed to low sequencing coverage, no clear association is observed in this case, suggesting that the early divergence of these cells may reflect genuine biological differentiation rather than technical noise.

A similar differentiation process was observed in Batch2 data (Fig. 7), as evidenced by the mapping of most CYT1 progenitor cells prior to the branching event (Fig. 7b). These results suggest that our phylogenetic analysis can classify cells solely based on variant call information and show good agreement with cell classifications based on expression profiling. More importantly, it is possible to align the progenitor CYT1 cells in a temporal order, which is difficult to achieve using pseudotime analysis alone without additional analysis.^108–110^

Pavličev et al. reported that their samples contained 2 putatively maternal DCs. However, our results suggest that the putative DCs appear to be differentiated from fetal CYT cells or stem cells of CYT (Figs. 6a and 7a). To further examine this issue, we reanalyzed single-cell expression data using t-SNE and identified 3 clusters. Both putative DCs (claimed by Pavličev et al.) belong to the same cluster (data not shown). Therefore, our analyses, which incorporated both expression and variant data, suggest that these cells are more similar to CYT cells than to any other cell type. In fact, the expression of critical DC-characteristic genes, such as *CLEC4C*, *THBD*, *CD1C*, *CD80*, *IL10*, and *IL12B*, was not found in these cells.^100^

Note that these cells were collected from the utero-placental interface.^100^ Because of the close proximity of the maternal and fetal tissues, there is a possibility that some of the sampled cells could be of fetal origin.

**Fig. 8.**
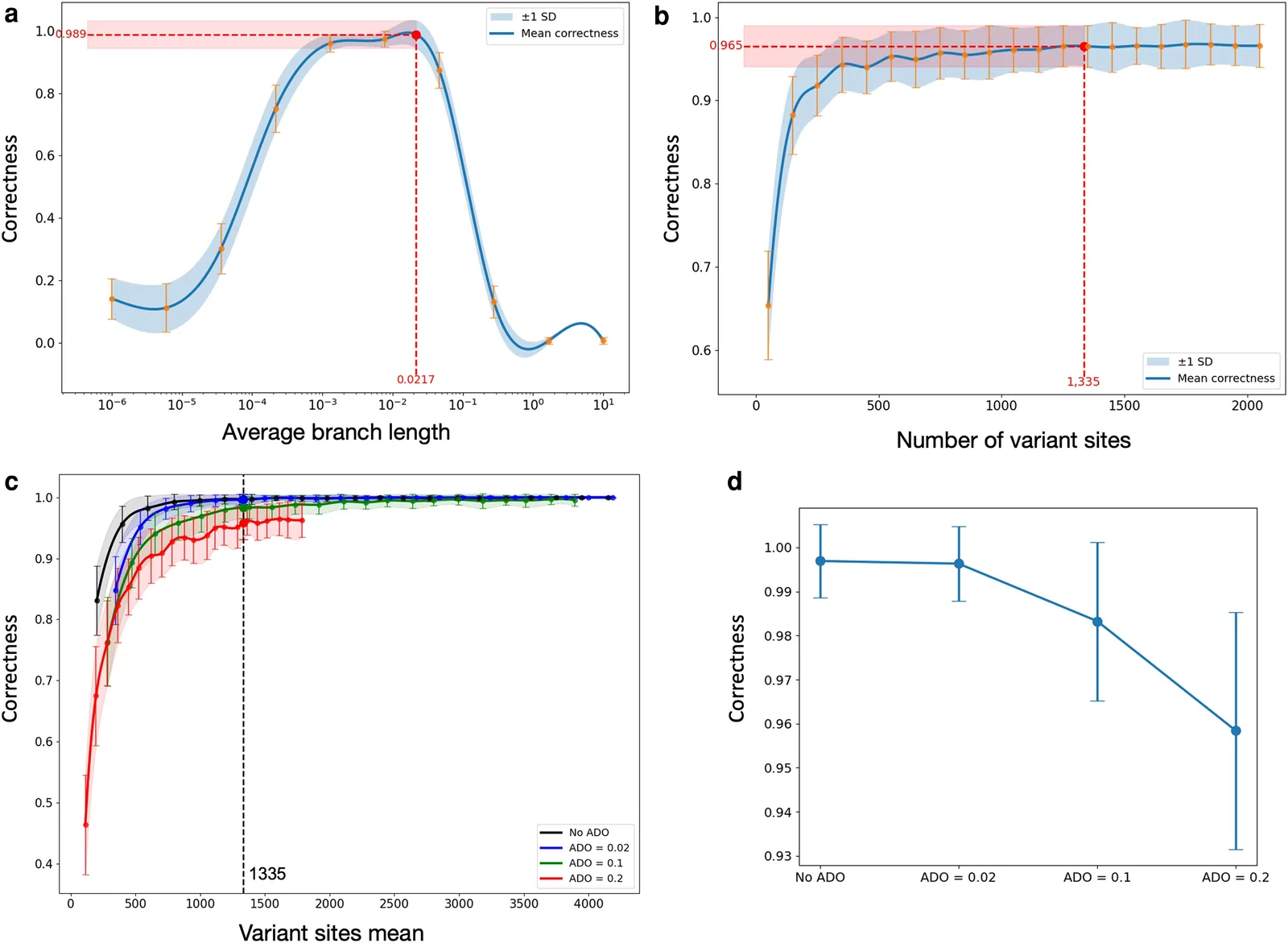
Theoretical simulation–based expectations of phylogenetic tree reconstruction accuracy and their correspondence to empirical real-data analysis results under various branch lengths, segregating-site counts, and allelic dropout (ADO) conditions. (a) Relationship between average branch length and phylogenetic tree reconstruction accuracy. The *x*-axis represents the average branch length on a logarithmic scale, and the *y*-axis indicates reconstruction correctness measured as 1−RF distance. The blue curve represents smoothed mean correctness values, while the shaded region indicates ±1 standard deviation. Red dashed lines indicate the expected reconstruction accuracy at an average branch length of 0.0217, corresponding to the estimated branch length derived from the real-data analysis (Batch 1: 53 cells). (b) Relationship between observed variant-site count and phylogenetic tree reconstruction accuracy. The *x*-axis represents the number of observed variant sites, and the *y*-axis indicates reconstruction correctness. The blue curve and shaded region represent smoothed mean correctness values and ±1 standard deviation, respectively. Red dashed lines indicate the estimated correctness corresponding to 1,335 observed variant sites, matching the value estimated from the empirical dataset. (c) Relationship between observed variant-site count and phylogenetic tree reconstruction accuracy under different allelic dropout (ADO) conditions. Black, blue, green, and red curves correspond to No ADO (ie, no noise), ADO = 0.02, ADO = 0.1, and ADO = 0.2, respectively. Solid curves represent smoothed mean correctness values, and shaded regions indicate ±1 standard deviation. The vertical dashed line indicates 1,335 observed variant sites, corresponding to the empirical estimate used for comparison with the real dataset. (d) Estimated phylogenetic tree reconstruction correctness at 1,335 observed variant sites under different ADO conditions. Points represent interpolated mean correctness values derived from the smoothed curves in panel (c), and error bars indicate interpolated standard deviations. Increasing ADO progressively reduces the expected reconstruction accuracy, although relatively high accuracy is maintained under moderate ADO levels.

The results demonstrate a close correspondence between theoretical simulation–based expectations and the empirical characteristics observed in the real scRNA-seq dataset. As shown in Fig. 8a, the empirically estimated average branch length from the real dataset (0.0217) falls near the high-accuracy region of the theoretical curve, corresponding to an expected correctness of ∼0.989. This result suggests that the empirical dataset contains sufficient phylogenetic signal for relatively accurate lineage reconstruction.

Figure 8b further demonstrates the relationship between the number of observed variant sites and reconstruction accuracy. Accuracy increases rapidly as the number of observed variant sites rises, eventually reaching a plateau near the maximal correctness. The empirical estimate of 1,335 observed variant sites corresponds to a predicted correctness of ∼0.965, again placing the real dataset within the theoretically favourable regime for lineage reconstruction. This agreement supports the consistency between the observed empirical mutation burden and the theoretical expectations derived from simulation.

Figure 8c shows that ADO progressively degrades reconstruction accuracy across all variant-site counts. Nevertheless, even under moderate ADO levels, reconstruction accuracy remains relatively high when sufficient variant sites are available. The empirical estimate of 1,335 variant sites intersects the curves within regions, maintaining high correctness across all tested ADO conditions. These results indicate that adequate phylogenetic signal can partially compensate for substantial scRNA-seq noise.

Finally, Fig. 8d summarizes the expected reconstruction correctness at 1,335 observed variant sites under different ADO conditions. Although increasing ADO systematically lowers expected accuracy, the reduction remains moderate up to ADO = 0.1, while stronger degradation becomes apparent at ADO = 0.2. Importantly, even under relatively high dropout conditions, the expected correctness remains above ∼0.95. Collectively, these results suggest that the empirical dataset lies within a parameter regime where accurate phylogenetic reconstruction is theoretically achievable despite substantial scRNA-seq–specific noise and sparsity.

### 3.8. Pseudotime analysis

We compared the cells aligned in a temporal order by RTCA with those of pseudotime analysis.^108–110^

**Fig. 9.**
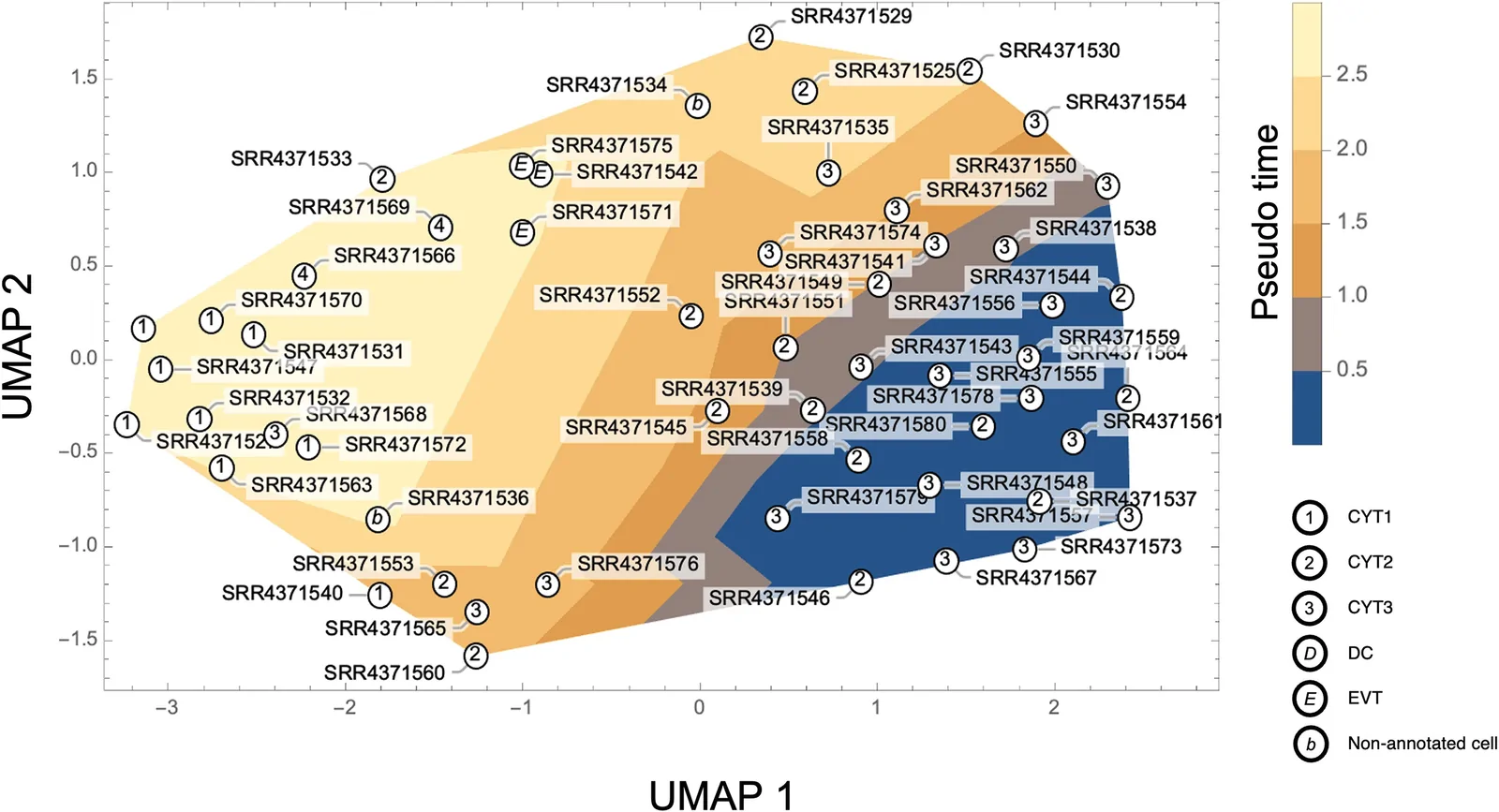
Results of pseudotime analysis for SRP090944 Batch1. Pseudotime values are visualized as colour gradients over a UMAP embedding, with contours generated using the ListContourPlot function in Mathematica,^111^ using interpolation order 3. The colour legend represents inferred pseudotime, from early (dark blue) to late (light yellow) stages. Due to limitations in Mathematica's labelling algorithm, not all cell IDs are shown. Cell type annotations are as follows: 1—Intravillous cytotrophoblast (CYT1); 2—CYT2; 3—CYT3; D—putative decidual dendritic cell (DC); E—extravillous cytotrophoblast (EVT); b—non-annotated cell. See also Figs. 6 and 7 for reference.

Figure 9 shows the results of pseudotime analysis of Batch1 represented by contours created by a function of *Mathematica*.^111^ The cells mapped onto the pseudotime contours are marked by the letters or numbers corresponding to the cell types classified by Pavličev et al.^100^ Their classification is more consistent with the result of our RTCA than the result of pseudotime analysis. The results of both pseudotime and RTCA analyses were generally consistent with the direction of differentiation. Sixteen cells had pseudotime values greater than 2.5, ten of which corresponded to CYT1 progenitor cells. Cells differentiated from CYT1 cells, ie, CYT2 and CYT3, are detected in regions with lower pseudotime values, suggesting again that these cells are temporally distant from CYT1 cells in the cell lineage. Although EVT cells are located at close proximity to CYT1 cells on the pseudotime scale, our RTCA indicates that these cells are not closely related to CYT1 cells, which is consistent with previous biological knowledge.^125^ Therefore, our RTCA can yield the same lineage trajectories but at a higher resolution compared to the pseudotime analysis.

### 3.9. Quantifying alignment–tree inconsistency under the ISM

We applied the tree-aware consistency analysis to the real scRNA-seq alignment (Batch 1 data) and the corresponding minimum evolution (ME) phylogenetic tree. After preprocessing to remove invariant sites and columns with excessive missingness (>20% of cells), a total of 1,335 alignment columns were retained for analysis. Among these, 525 sites (39.3%) violated the infinite sites model (ISM) consistency criterion under the missing-data–tolerant definition, yielding an empirical inconsistency rate of $\hat{\epsilon }=0.393.$

Under the adopted criterion, a site was considered consistent if all observed derived alleles formed a single clade in the inferred tree, and no observed ancestral alleles were present in that clade, with missing values permitted in either region. Consequently, the observed inconsistency rate reflects a combination of factors, including ADO and false-positive variant calls inherent to scRNA-seq data, alignment and variant-calling artefacts, finite-sites effects leading to homoplasy, and residual errors in the estimated phylogenetic tree.

Notably, despite the substantial technical noise characteristic of scRNA-seq, approximately two-thirds of the analyzed sites (60.7%) were compatible with the inferred lineage tree under this relaxed definition. This level of compatibility indicates that the estimated tree captures a meaningful fraction of the underlying mutational structure in the data, while also highlighting the prevalence of site-level deviations from idealized infinite sites assumptions in real single-cell transcriptomic datasets.

## 4. Discussion

This study presents a simple direct phylogenetic signal framework^45,127,128^ for inferring cell lineage trees from somatic variants detected exclusively in scRNA-seq data from normal tissues. In contrast to genotype-mediated inference frameworks^42–44^ that focus on cancer-associated mutations, we emphasize the phylogenetic signal embedded in the sparse variant calls of normal cells. Our results demonstrate that lineage trees can be reconstructed from such sparse scRNA-seq variants and that the inferred trees closely recapitulate established biological differentiation pathways.

Importantly, RTCA inherently integrates lineage information with corresponding gene expression profiles, while these data types are typically analyzed separately. This orthogonal integration provides deeper insights into the biological system under investigation.

RTCA has a nuanced yet distinctive characteristic compared with existing lineage-inference methods based solely on nuclear and/or mitochondrial scRNA-seq data, such as PhylinSic^29^ and LINEAGE.^31^ These methods are genotype-mediated inference framework,^42–44^ in that lineage inference is mediated through per-cell genotype representations, whereas RTCA is direct phylogenetic signal framework,^45,127,128^ accumulating phylogenetic signals across cells without introducing an explicit per-cell genotype layer.

To make this distinction explicit, we formalized the posterior distributions under genotype-mediated inference and direct phylogenetic signal frameworks for lineage tree reconstruction from scRNA-seq mutation data. In summary, although both approaches may operate on cell-indexed representations, genotype-mediated inference frameworks infer trees through a latent per-cell genotype layer G (Eqs. 16–18), whereas RTCA performs inference directly on observed site patterns with explicit missingness (Eqs. 19–21). The resulting approximations to the full marginal likelihood (Eq. 21) therefore diverge most strongly under the extreme missingness characteristic of normal tissues (see Materials and Methods for details).

**Fig. 10.**
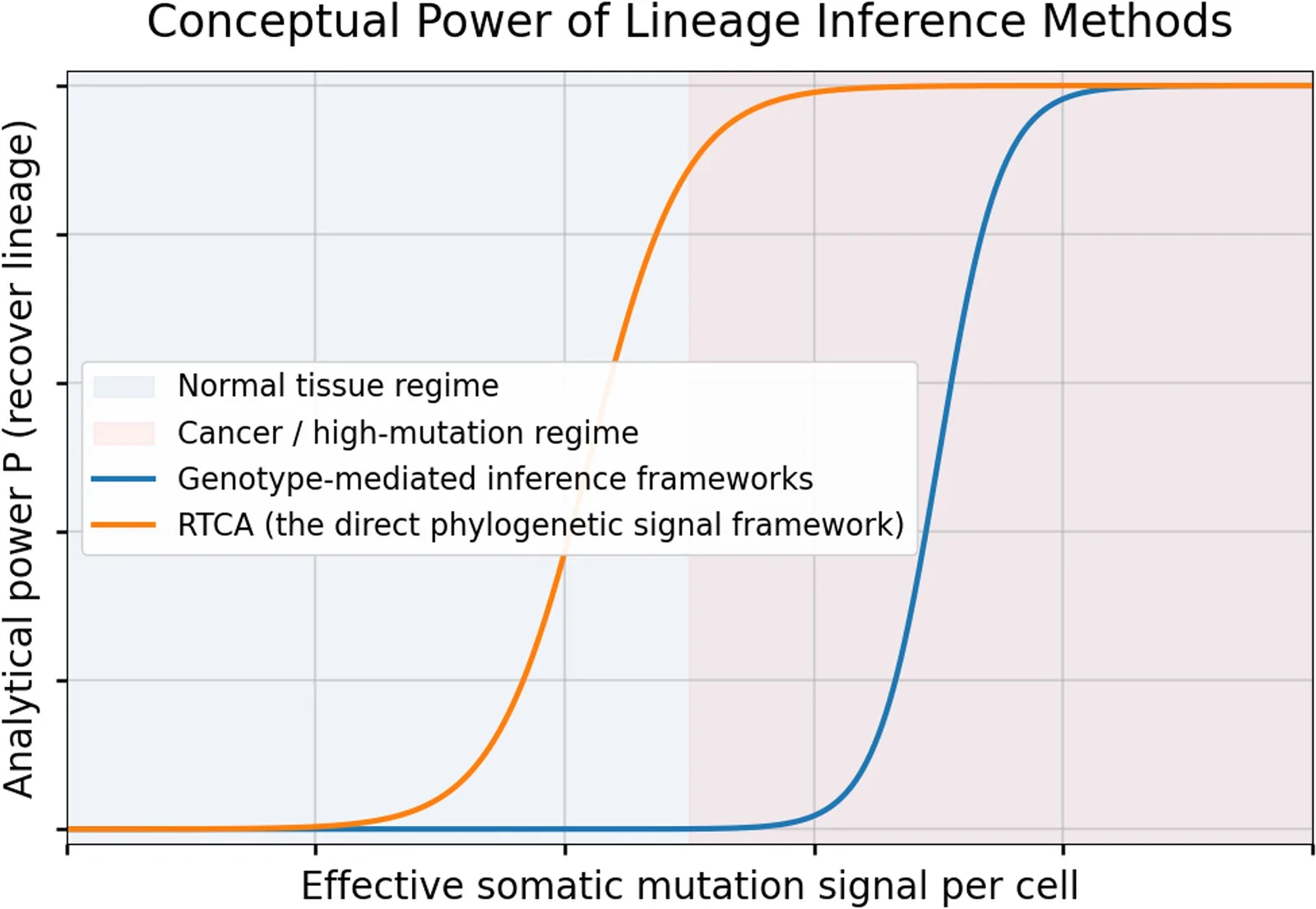
Conceptual power comparison of lineage inference methods across mutation regimes. Schematic illustration of the analytical power *P* to recover lineage structure as a function of the effective somatic mutation signals per cell (log scale). The blue curve represents barcode-based, cell-genotype–centric lineage inference methods, whose power rises sharply only when mutation burden per cell is high. The orange curve represents RTCA, a direct phylogenetic signal framework approach that aggregates site patterns across cells and thus achieves substantial power at much lower mutation signals. Shaded regions indicate typical biological regimes: the normal tissue regime (low mutation burden) and the cancer/high-mutation regime (elevated mutation burden). The horizontal separation between curves highlights the conceptual advantage of direct phylogenetic signal framework methods in low-mutation, non-cancerous tissues, where genotype-mediated inference framework approaches lack sufficient signals.

Figure 10 illustrates the schematic relationship between genotype-mediated inference frameworks (existing methods) and the direct phylogenetic signal framework represented by RTCA. While the 2 approaches may converge under regimes with higher mutation burdens in normal tissues, the mutation burden is extremely low (4 to 100 times lower than in cancerous tissues^9^). Under such conditions, most cells have zero or near-zero callable sites, rendering per-cell genotype inference ill-posed.

In genotype-mediated inference framework pipelines, variation is represented as latent per-cell genotypes defined relative to a reference genome, whereas in RTCA, the primary data object is the observed site pattern, which encodes mutual variability across cells. Lineage inference in RTCA therefore depends on relational consistency among cells rather than on reference-anchored genotype inference.

The majority of existing methods have been developed for cancer tissue samples, with a primary focus on accurately detecting driver mutations that contribute to cancer development and progression.^129^ These cancer-oriented paradigm may not be suitable for retrospective cell lineage inference, where the detection of a comprehensive set of neutral somatic mutations—which serve as phylogenetic markers of cell division history—is essential for reconstructing accurate lineage relationships.^130^ In this context, the sensitivity to low-frequency, non-functional variants and the ability to minimize false positives without prior knowledge of mutation hotspots become critical challenges.

A potential limitation of methods that reconstruct cell lineage trees using endogenous mutations detected exclusively in scRNA-seq data, including RTCA, is that tree topology is inferred solely from phylogenetic signals embedded in SNVs derived from scRNA-seq data. These signals can be obscured by random noise introduced by sparse coverage,^22^ ADO,^23^ amplification bias,^24^ RNA editing,^25^ technical noise,^26^ expression bias,^131^ and the absence of matched DNA data.^27^ Nevertheless, we demonstrate the theoretical plausibility of reconstructing reliable phylogenies despite the phylogenetic noise introduced by these factors and show that a sufficient number of segregating sites can still be obtained for robust inference.

In the context of normal tissue development, it is essential to consider how frequently observable somatic mutations arise and whether the segregating sites we observe are consistent with theoretical expectations. In normal human cells, the best-supported upper range is approximately 1 to 8 SNVs per genome per cell division (corresponding to ∼0.3 to 2.7 × 10^−9^ mutations per base per division).^9,10^ Intuitively, this mutation rate appears too low to allow reconstruction of a reliable binary lineage tree. However, our results align with the theoretical expectation, as shown below.

We assume that the total mitoses in placenta are $3\times 10^{13}$^62–65^ (see Cell Population Kinetics in Supplementary for detail), the final cell count is $5\times 10^{11}$, founder (30 cells)→term ($5\times 10^{11}$ cells) minimum doublings is $\log_{2}\;(5\times 10^{11}/30)\approx 34$, and implied depth per surviving lineage is the turnover factor $(3\times 10^{13}/5\times 10^{11})=60$.

Setting n=50 cells, h=2,040 divisions/tip, and $E[T_{50}]\approx 456,879$ divisions—and targeting S=1,000 segregating sites, the required per-division mutation rate and per-cell burden are


(23) $$r_{\mathrm{req}}=\frac{S\cdot 3\times 10^{9}}{E[T_{n}]\cdot L_{\mathrm{align}}}\;\left(\mathrm{SNVs}/\mathrm{division}\right)$$

and


(24) $$M_{\mathrm{cell}}=r_{\mathrm{req}}\cdot h\;(\mathrm{SNVs}\;\mathrm{per}\;\mathrm{cell}),$$

where $M_{\mathrm{cell}}$ is the required true burden per cell (SNVs per genome at term). See Supplementary material for detail.

In our analysis, approximately 0.5% of the genome was aligned to the scRNA-seq data (see Figure S4), and the across-cell intersection ($L_{\mathrm{align}}$) was typically ≤ 15 Mb. Under this condition, the required per-division mutation rate and per-cell mutation burden were estimated to be 0.44 SNVs per division and 893 SNVs per cell, respectively. This implies that achieving approximately 1,000 segregating sites across ∼50 human placental cells would require a per-division mutation rate of 0.44 SNVs per genome, which is slightly below the reported range of ∼1 to 8 SNVs per genome per division. Under these assumptions, this target is therefore theoretically attainable.

In this study, we reconstructed bifurcating trees to represent cell lineage relationships. However, it is important to note that tree reconstruction accuracy is significantly influenced by the evolutionary distances among sequences.^132^ In our simulations, we set the average branch length of the randomly generated truth trees such that each variant site was expected to harbour at least one mutation. As long as the simulation parameters remained within this empirically determined “sweet spot” region (Fig. 3), reliable binary trees could be reconstructed.

It is noteworthy that the theoretical branch length for 1,000 OTUs is $1.38\times 10^{-4}$ –$3.71\times 10^{-3}$ (approximately 0.1 to 1 mutation per branch, or $∼10^{-3}$ mutations per site for 1,000 sampled cells; see Fig. 1). These values fall within the simulated “sweet-spot” region described in the Materials and Methods. This finding indicates that the theoretical expectation can indeed lead to reliable tree reconstruction using the ME method.

Nevertheless, the minimum number of variant sites required to accurately reconstruct a bifurcating tree in our context remains an open question. To address this, we performed simulations to evaluate the impact of the variant site count on reconstruction accuracy. The results showed that with 300 variant sites, a binary tree with over 90% accuracy could be reconstructed for 50 and 100 cells (Fig. 4a-b). Likewise, with 400 variant sites, a similar level of accuracy was achievable for 1,000 cells (Fig. 4c). These findings highlight the scalability and robustness of our method.

One might argue that our analysis addresses only false negatives in segregating-site detection, without explicitly considering false positives. To address this concern, it is necessary to examine several nuanced yet critical features of scRNA-seq analysis.

scRNA-seq introduces substantially more noise than genomic DNA sequencing due to reverse transcription errors, low input RNA, amplification biases, and the inherent complexity of the transcriptome.^26,70,119^ The combined effective mismatch error rate in aligned scRNA-seq reads is approximately 10^−3^ per base (0.1%), a level consistently reported across platforms and 100 to 1,000 times higher than that observed in bulk genomic DNA sequencing. This elevated error rate has led to the prevailing assumption that detecting somatic mutations solely from scRNA-seq data is virtually impossible.^26,70,119^ However, this view overlooks a key conceptual distinction: whereas bulk DNA sequencing provides population-averaged information, scRNA-seq captures the transcriptomes of individual cells, thereby preserving cell-specific variation that can be exploited to denoise raw mutational signals.^133–135^

Random mismatches introduced during reverse transcription, amplification, or sequencing arise independently across molecules and across cells and therefore do not recur at the same genomic position with consistent allele identity or frequency.^24,136^ In contrast, bona fide somatic mutations generate reproducible alternative alleles across multiple transcripts and across subsets of clonally related cells, yielding coherent recurrence patterns that are exceedingly unlikely to arise from stochastic noise.^133^ At the site level, random sequencing errors tend to average towards zero variant allele frequency, whereas true somatic mutations maintain stable, non-zero allele fractions across the cells that harbour them.^137^ Moreover, transcription amplifies each mutated allele into multiple RNA molecules, increasing the probability of detecting the corresponding variant even when genomic DNA coverage is low. Recent studies have empirically demonstrated that, with appropriate filtering strategies—including recurrence-based detection, allele-balance modelling, exclusion of known RNA-editing sites, and phylogenetic consistency constraints—somatic mutations can be reliably recovered from scRNA-seq data.^119,134^

Taken together, these findings indicate that, despite the high background error rate, scRNA-seq retains sufficient structured information to enable the detection of somatic mutations and the reconstruction of lineage relationships. Therefore, with current analytical approaches, false-positive detection of somatic mutations from scRNA-seq data is no longer a major concern. The remaining open question is whether false negatives can be effectively addressed, particularly in normal cell populations that harbour only sparse somatic variants. Our analyses provide a clear yes answer to this question.

**Fig. 11.**
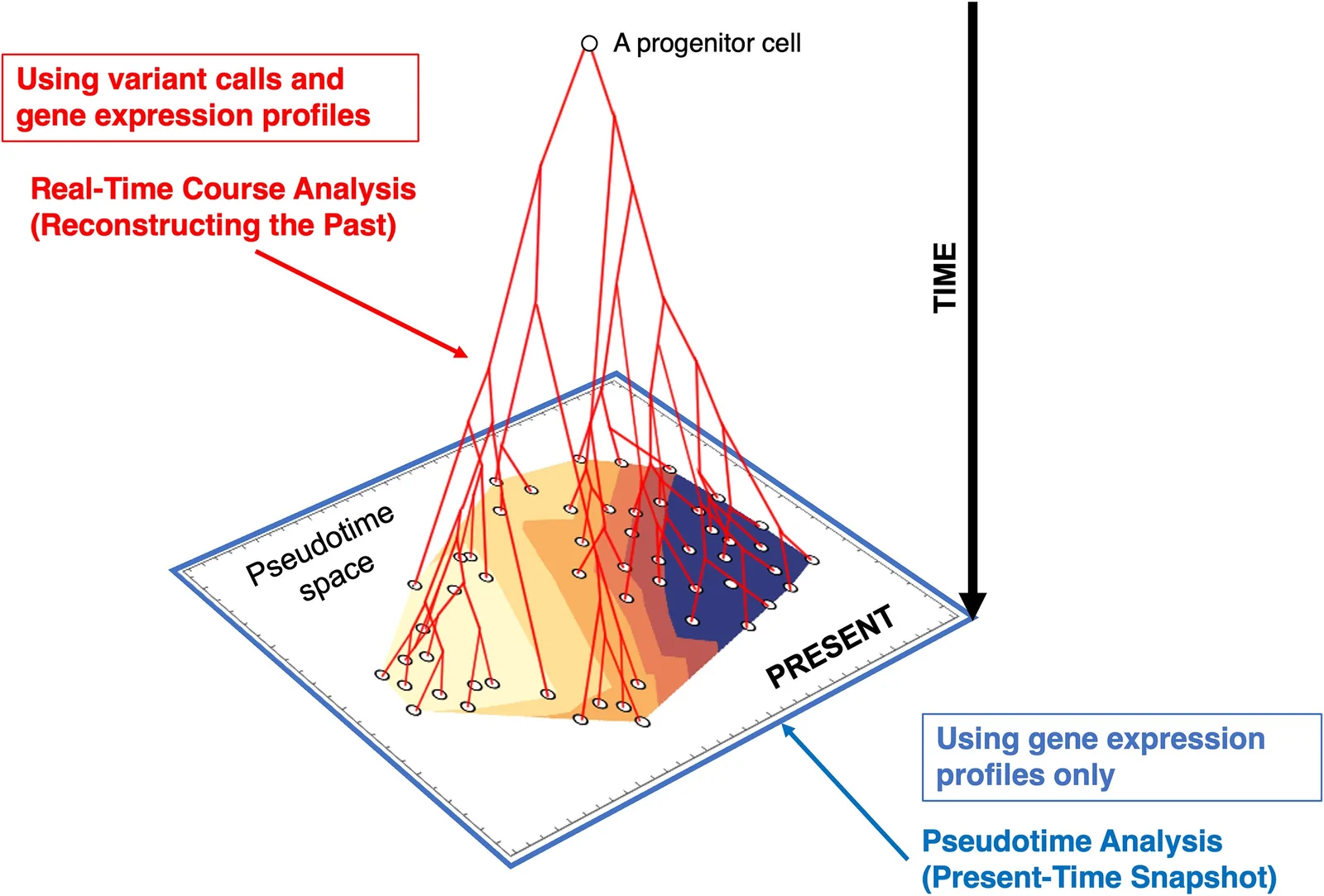
Conceptual distinction between real-time course analysis (RTCA) and pseudotime analysis. This schematic illustrates 2 complementary approaches to modelling cellular dynamics. Pseudotime analysis (blue, bottom layer) infers the progression of cell states based on gene expression data captured at a single time point—effectively a snapshot of the present. Cells are ordered along a trajectory derived from transcriptomic similarity, but without direct historical information. In contrast, real-time course analysis (RTCA, shown in red) uses variant calls to reconstruct the actual lineage history of cells—offering a retrospective view of how current cell states emerged over time. This approach provides a tree-like reconstruction of the past, tracing back from present cells to their common ancestors. Importantly, RTCA inherently integrates lineage information with gene expression dynamics over time, while pseudotime analysis treats the 2 data types separately. This distinction emphasizes RTCA’s advantage in modelling true developmental processes when somatic variants are available.

Because somatic mutations accumulate over time, our RTCA framework enables the reconstruction of cell lineages back to the progenitor cells, allowing the inference of a cellular time course, including unobservable past events. Notably, the RTCA results were more consistent with known biological processes than those obtained from pseudotime analysis. The key distinction is that RTCA, based on mutational data, can infer linear and temporal relationships between cells in terms of the number of accumulated mutations, whereas pseudotime analysis infers only non-linear and spatial relationships within a single time frame (Fig. 11). To overcome this limitation, pseudotime approaches may need to incorporate splicing kinetics, such as RNA velocity,^138^ to enhance temporal resolution.

In conclusion, our RTCA is highly effective and affordable, especially in human biology, in which experimental manipulation is strictly restricted. It has great potential to delineate normal cell trajectories during development and to interpret high-dimensional gene expression data. Furthermore, the biological significance of somatic mutations provides insight into a new perspective of ‘evolution’ within an individual.

## Supplementary Material

dsag007_Supplementary_Data

## Data Availability

The data supporting the findings of this study are divided into 2 sets: Batch1 (54 cells) and Batch2 (33 cells), both derived from the Series GSE87720 in the Gene Expression Omnibus (GEO) database. The corresponding sequencing data are accessible under the accession numbers SRR4371525 (GSM2339239) to SRS1732319 (SRX2225328).

## Program availability

The source code is available on GitHub at: [TBA].

## References

[1] Kelly DP . Ageing theories unified. Nature. 2011:470:342. 10.1038/nature09896 21307852

[2] Kennedy SR, Loeb LA, Herr AJ. Somatic mutations in aging, cancer and neurodegeneration. Mech Ageing Dev. 2012:133:118–126. 10.1016/j.mad.2011.10.009 22079405 PMC3325357

[3] Morley AA . The somatic mutation theory of ageing. Mutat Res. 1995:338:19–23. 10.1016/0921-8734(95)00007-S 7565875

[4] Abeliovich A et al On somatic recombination in the central nervous system of transgenic mice. Science. 1992:257:404–410. 10.1126/science.1631561 1631561

[5] Yizhak K et al RNA sequence analysis reveals macroscopic somatic clonal expansion across normal tissues. Science. 2019:364:eaaw0726. 10.1126/science.aaw0726 PMC7350423 31171663

[6] Ju YS et al Somatic mutations reveal asymmetric cellular dynamics in the early human embryo. Nature. 2017:543:714–718. 10.1038/nature21703 28329761 PMC6169740

[7] Tomasetti C, Vogelstein B, Parmigiani G. Half or more of the somatic mutations in cancers of self-renewing tissues originate prior to tumor initiation. Proc Natl Acad Sci U S A. 2013:110:1999. 10.1073/pnas.1221068110 23345422 PMC3568331

[8] Lynch M . Rate, molecular spectrum, and consequences of human mutation. Proc Natl Acad Sci U S A. 2010:107:961. 10.1073/pnas.0912629107 20080596 PMC2824313

[9] Werner B et al Measuring single cell divisions in human tissues from multi-region sequencing data. Nat Commun. 2020:11:1035. 10.1038/s41467-020-14844-6 32098957 PMC7042311

[10] Milholland B et al Differences between germline and somatic mutation rates in humans and mice. Nat Commun. 2017:8:15183. 10.1038/ncomms15183 28485371 PMC5436103

[11] Jonsson H et al Differences between germline genomes of monozygotic twins. Nat Genet. 2021:53:27–34. 10.1038/s41588-020-00755-1 33414551

[12] García-Nieto PE, Morrison AJ, Fraser HB. The somatic mutation landscape of the human body. Genome Biol. 2019:20:298. 10.1186/s13059-019-1919-5 31874648 PMC6930685

[13] Rozhok AI, DeGregori J. Toward an evolutionary model of cancer: considering the mechanisms that govern the fate of somatic mutations. Proc Natl Acad Sci U S A. 2015:112:8914–8921. 10.1073/pnas.1501713112 26195756 PMC4517250

[14] Woodworth MB, Girskis KM, Walsh CA. Building a lineage from single cells: genetic techniques for cell lineage tracking. Nat Rev Genet. 2017:18:230. 10.1038/nrg.2016.159 28111472 PMC5459401

[15] Oota S . Somatic mutations—evolution within the individual. Methods. 2020:176:91–98. 10.1016/j.ymeth.2019.11.002 31711929

[16] Davenport CB . The personality, heredity and work of charles otis whitman, 1843–1910. Am Nat. 1917:51:5–30. 10.1086/279584

[17] McGranahan N, Swanton C. Clonal heterogeneity and tumor evolution: past, present, and the future. Cell. 2017:168:613–628. 10.1016/j.cell.2017.01.018 28187284

[18] Altrock P, Liu L, Michor F. The mathematics of cancer: integrating quantitative models. Nat Rev Cancer. 2015:15:730–745. 10.1038/nrc4029 26597528

[19] Kebschull JM, Zador AM. Cellular barcoding: lineage tracing, screening and beyond. Nat Methods. 2018:15:871–879. 10.1038/s41592-018-0185-x 30377352

[20] Wang Y, Navin NE. Advances and applications of single-cell sequencing technologies. Mol Cell. 2015:58:598–609. 10.1016/j.molcel.2015.05.005 26000845 PMC4441954

[21] Tam PPL, Ho JWK. Cellular diversity and lineage trajectory: insights from mouse single cell transcriptomes. Development. 2020:147:dev179788. 10.1242/dev.179788 31980483

[22] Tang F et al mRNA-Seq whole-transcriptome analysis of a single cell. Nat Methods. 2009:6:377–382. 10.1038/nmeth.1315 19349980

[23] Qiu P . Embracing the dropouts in single-cell RNA-seq analysis. Nat Commun. 2020:11:1169. 10.1038/s41467-020-14976-9 32127540 PMC7054558

[24] Islam S et al Quantitative single-cell RNA-seq with unique molecular identifiers. Nat Methods. 2014:11:163–166. 10.1038/nmeth.2772 24363023

[25] Kleinman CL, Majewski J. Comment on “Widespread RNA and DNA sequence differences in the human transcriptome”. Science. 2012:335:1302. 10.1126/science.1209658 22422962

[26] Brennecke P et al Accounting for technical noise in single-cell RNA-seq experiments. Nat Methods. 2013:10:1093–1095. 10.1038/nmeth.2645 24056876

[27] Vu TN et al Cell-level somatic mutation detection from single-cell RNA sequencing. Bioinformatics. 2019:35:4679–4687. 10.1093/bioinformatics/btz288 31028395 PMC6853710

[28] Martincorena I, Campbell PJ. Somatic mutation in cancer and normal cells. Science. 2015:349:1483–1489. 10.1126/science.aab4082 26404825

[29] Liu X et al Phylogenetic inference from single-cell RNA-seq data. Sci Rep. 2023:13:12854. 10.1038/s41598-023-39995-6 37553438 PMC10409753

[30] Moravec JC, Lanfear R, Spector DL, Diermeier SD, Gavryushkin A. Testing for phylogenetic signal in single-cell RNA-Seq data. J Comput Biol. 2023:30:518–537. 10.1089/cmb.2022.0357 36475926 PMC10125402

[31] Lin L et al LINEAGE: label-free identification of endogenous informative single-cell mitochondrial RNA mutation for lineage analysis. Proc Natl Acad Sci U S A. 2022:119:e2119767119. 10.1073/pnas.2119767119 35086932 PMC8812554

[32] Lareau CA et al Massively parallel single-cell mitochondrial DNA genotyping and chromatin profiling. Nat Biotechnol. 2021:39:451–461. 10.1038/s41587-020-0645-6 32788668 PMC7878580

[33] Kwok AWC et al MQuad enables clonal substructure discovery using single cell mitochondrial variants. Nat Commun. 2022:13:1205. 10.1038/s41467-022-28845-0 35260582 PMC8904442

[34] Yu X et al MitoTracer facilitates the identification of informative mitochondrial mutations for precise lineage reconstruction [preprint]. bioRxiv 2023;568285. 10.1101/2023.11.22.568285 PMC12184895 40549685

[35] Wang M, Deng W, Samuels DC, Zhao Z, Simon LM. MitoTrace: a computational framework for analyzing mitochondrial variation in single-cell RNA sequencing data. Genes (Basel). 2023:14:1222. 10.3390/genes14061222 37372402 PMC10298143

[36] Sun W, van Ginneken D, Perié L. scMitoMut for calling mitochondrial lineage-related mutations in single cells. Brief Bioinform. 2024:26:bbaf072. 10.1093/bib/bbaf072 PMC11878546 40036721

[37] McCarthy DJ et al Cardelino: computational integration of somatic clonal substructure and single-cell transcriptomes. Nat Methods. 2020:17:414–421. 10.1038/s41592-020-0766-3 32203388

[38] Jun S-H et al Reconstructing clonal tree for phylo-phenotypic characterization of cancer using single-cell transcriptomics. Nat Commun. 2023:14:982. 10.1038/s41467-023-36202-y 36813776 PMC9946941

[39] Edrisi M, Huang X, Ogilvie HA, Nakhleh L. Accurate integration of single-cell DNA and RNA for analyzing intratumor heterogeneity using MaCroDNA. Nat Commun. 2023:14:8262. 10.1038/s41467-023-44014-3 38092737 PMC10719311

[40] Campbell KR et al Clonealign: statistical integration of independent single-cell RNA and DNA sequencing data from human cancers. Genome Biol. 2019:20:54. 10.1186/s13059-019-1645-z 30866997 PMC6417140

[41] Maslov AY et al Single-molecule, quantitative detection of low-abundance somatic mutations by high-throughput sequencing. Sci Adv. 2022:8:eabm3259. 10.1126/sciadv.abm3259 PMC8993124 35394831

[42] Felsenstein J . Evolutionary trees from DNA sequences: a maximum likelihood approach. J Mol Evol. 1981:17:368–376. 10.1007/BF01734359 7288891

[43] Yang Z . Maximum likelihood phylogenetic estimation from DNA sequences with variable rates over sites: approximate methods. J Mol Evol. 1994:39:306–314. 10.1007/BF00160154 7932792

[44] Jahn K, Kuipers J, Beerenwinkel N. Tree inference for single-cell data. Genome Biol. 2016:17:86. 10.1186/s13059-016-0936-x 27149953 PMC4858868

[45] Felsenstein J . Inferring phylogenies. Sinauer Associates; 2004.

[46] Yang Z, Rannala B. Molecular phylogenetics: principles and practice. Nat Rev Genet. 2012:13:303–314. 10.1038/nrg3186 22456349

[47] Hinton G, Roweis S. Stochastic Neighbor Embedding (SNE). In: Anselin L. editor, An introduction to spatial data science with GeoDa. Chapman and Hall/CRC; 2003:2:833–840.

[48] McInnes L, Healy J, Melville J. UMAP: Uniform Manifold Approximation and Projection for Dimension Reduction [preprint]. arXiv 2020;arXiv:1802.03426. 10.48550/arXiv.1802.03426

[49] Kobak D, Linderman GC. Initialization is critical for preserving global data structure in both t-SNE and UMAP. Nat Biotechnol. 2021:39:156–157. 10.1038/s41587-020-00809-z 33526945

[50] Ota S, Li W-H. NJML: a hybrid algorithm for the neighbor-joining and maximum-likelihood methods. Mol Biol Evol. 2000:17:1401–1409. 10.1093/oxfordjournals.molbev.a026423 10958856

[51] Watterson GA . On the number of segregating sites in genetical models without recombination. Theor Popul Biol. 1975:7:256–276. 10.1016/0040-5809(75)90020-9 1145509

[52] Hudson RR . Gene genealogies and the coalescent process. In: Oxford surveys in evolutionary biology (pp. 1–44). Vol. 7: Oxford Academic; 1990.

[53] Kimura M . The number of heterozygous nucleotide sites maintained in a finite population due to steady flux of mutations. Genetics. 1969:61:893–903. 10.1093/genetics/61.4.893 5364968 PMC1212250

[54] Tajima F . Infinite-allele model and infinite-site model in population genetics. J Genet. 1996:75:27. 10.1007/BF02931749

[55] Liu Y et al Single-cell RNA-seq reveals the diversity of trophoblast subtypes and patterns of differentiation in the human placenta. Cell Res. 2018:28:819–832. 10.1038/s41422-018-0066-y 30042384 PMC6082907

[56] Vanea C et al Mapping cell-to-tissue graphs across human placenta histology whole slide images using deep learning with HAPPY. Nat Commun. 2024:15:2710. 10.1038/s41467-024-46986-2 38548713 PMC10978962

[57] Araten DJ et al A quantitative measurement of the human somatic mutation rate. Cancer Res. 2005:65:8111–8117. 10.1158/0008-5472.CAN-04-1198 16166284

[58] Ng SB et al Targeted capture and massively parallel sequencing of 12 human exomes. Nature. 2009:461:272–276. 10.1038/nature08250 19684571 PMC2844771

[59] Coffey AJ et al The GENCODE exome: sequencing the complete human exome. Eur J Hum Genet. 2011:19:827–831. 10.1038/ejhg.2011.28 21364695 PMC3137498

[60] Pertea M . The human transcriptome: an unfinished story. Genes (Basel). 2012:3:344–360. 10.3390/genes3030344 22916334 PMC3422666

[61] Schadt EE et al A comprehensive transcript index of the human genome generated using microarrays and computational approaches. Genome Biol. 2004:5:R73. 10.1186/gb-2004-5-10-r73 15461792 PMC545593

[62] Mayhew TM . Turnover of human villous trophoblast in normal pregnancy: what do we know and what do we need to know? Placenta. 2014:35:229–240. 10.1016/j.placenta.2014.01.011 24529666

[63] Huppertz B et al Hypoxia favours necrotic versus apoptotic shedding of placental syncytiotrophoblast into the maternal circulation. Placenta. 2003:24:181–190. 10.1053/plac.2002.0903 12566245

[64] Askelund KJ, Chamley LW. Trophoblast deportation part I: review of the evidence demonstrating trophoblast shedding and deportation during human pregnancy. Placenta. 2011:32:716–723. 10.1016/j.placenta.2011.07.081 21855136

[65] Coorens THH et al Inherent mosaicism and extensive mutation of human placentas. Nature. 2021:592:80–85. 10.1038/s41586-021-03345-1 33692543 PMC7611644

[66] Bloise E et al Altered umbilical cord blood nutrient levels, placental cell turnover and transporter expression in human term pregnancies conceived by Intracytoplasmic Sperm Injection (ICSI). Nutrients. 2021:13:2587. 10.3390/nu13082587 34444747 PMC8399441

[67] Steel M, McKenzie A. Properties of phylogenetic trees generated by yule-type speciation models. Math Biosci. 2001:170:91–112. 10.1016/S0025-5564(00)00061-4 11259805

[68] Kingman JFC . On the genealogy of large populations. J Appl Probab. 1982:19:27–43. 10.2307/3213548

[69] Kim JK, Kolodziejczyk AA, Ilicic T, Teichmann SA, Marioni JC. Characterizing noise structure in single-cell RNA-seq distinguishes genuine from technical stochastic allelic expression. Nat Commun. 2015:6:8687. 10.1038/ncomms9687 26489834 PMC4627577

[70] Ziegenhain C et al Comparative analysis of single-cell RNA sequencing methods. Mol Cell. 2017:65:631–643.e4. 10.1016/j.molcel.2017.01.023 28212749

[71] Liu F et al Systematic comparative analysis of single-nucleotide variant detection methods from single-cell RNA sequencing data. Genome Biol. 2019:20:242. 10.1186/s13059-019-1863-4 31744515 PMC6862814

[72] Spiro A, Shapiro E. Accuracy of answers to cell lineage questions Depends on single-cell genomics data quality and quantity. PLoS Comput Biol. 2016:12:e1004983. 10.1371/journal.pcbi.1004983 27295404 PMC4905655

[73] Ramaswami G, Li JB. RADAR: a rigorously annotated database of A-to-I RNA editing. Nucleic Acids Res. 2014:42:D109–D113. 10.1093/nar/gkt996 24163250 PMC3965033

[74] Hangauer MJ, Vaughn IW, McManus MT. Pervasive transcription of the human genome produces thousands of previously unidentified long intergenic noncoding RNAs. PLoS Genet. 2013:9:e1003569. 10.1371/journal.pgen.1003569 23818866 PMC3688513

[75] Tajima F . Evolutionary relationship of DNA sequences in finite populations. Genetics. 1983:105:437–460. 10.1093/genetics/105.2.437 6628982 PMC1202167

[76] Wakeley J . Coalescent theory: an Introduction. Roberts & Company Publishers; 2009.

[77] Cheek D, Antal T. Genetic composition of an exponentially growing cell population. Stoch Process Their Appl. 2020:130:6580–6624. 10.1016/j.spa.2020.06.003

[78] Gunnarsson EB, Leder K, Foo J. Exact site frequency spectra of neutrally evolving tumors: a transition between power laws reveals a signature of cell viability. Theor Popul Biol. 2021:142:67–90. 10.1016/j.tpb.2021.09.004 34560155

[79] Tuyéras R . Category theory for genetics I: mutations and sequence alignments [preprint]. arXiv 2018;arXiv:1805.07002. 10.48550/arXiv.1805.07002

[80] Ashburner M et al Gene ontology: tool for the unification of biology. Nat Genet. 2000:25:25–29. 10.1038/75556 10802651 PMC3037419

[81] Thomas R . Boolean formalization of genetic control circuits. J Theor Biol. 1973:42:563–585. 10.1016/0022-5193(73)90247-6 4588055

[82] Juanes Cortés B et al Formalization of gene regulation knowledge using ontologies and gene ontology causal activity models. Biochim Biophys Acta Gene Regul Mech. 2021:1864:194766. 10.1016/j.bbagrm.2021.194766 34710644

[83] Karasavvas KA, Baldock R, Burger A. Bioinformatics integration and agent technology. J Biomed Inform. 2004:37:205–219. 10.1016/j.jbi.2004.04.003 15196484

[84] Sun X-L et al Stem cell competition driven by the Axin2-p53 axis controls brain size during murine development. Dev Cell. 2023:58:744–759.e11. 10.1016/j.devcel.2023.03.016 37054704

[85] Huerta-Cepas J, Serra F, Bork P. ETE 3: reconstruction, analysis, and visualization of phylogenomic data. Mol Biol Evol. 2016:33:1635–1638. 10.1093/molbev/msw046 26921390 PMC4868116

[86] Hasegawa M, Kishino H, Yano T-A. Dating of the human-ape splitting by a molecular clock of mitochondrial DNA. J Mol Evol. 1985:22:160–174. 10.1007/BF02101694 3934395

[87] Tamura K, Stecher G, Kumar S. MEGA11: molecular evolutionary genetics analysis version 11. Mol Biol Evol. 2021:38:3022–3027. 10.1093/molbev/msab120 33892491 PMC8233496

[88] Rzhetsky A, Nei M. A simple method for estimating and testing Minimum-evolution trees. Mol Biol Evol. 1992:9:945–945. 10.1093/oxfordjournals.molbev.a040771

[89] Tamura K, Nei M, Kumar S. Prospects for inferring very large phylogenies by using the neighbor-joining method. Proc Natl Acad Sci U S A. 2004:101:11030–11035. 10.1073/pnas.0404206101 15258291 PMC491989

[90] Robinson DF, Foulds LR. Comparison of phylogenetic trees. Math Biosci. 1981:53:131–147. 10.1016/0025-5564(81)90043-2

[91] Schiebinger G et al Optimal-transport analysis of single-cell gene expression identifies developmental trajectories in reprogramming. Cell. 2019:176:928–943.e22. 10.1016/j.cell.2019.01.006 30712874 PMC6402800

[92] Li JB et al Genome-wide identification of human RNA editing sites by parallel DNA capturing and sequencing. Science. 2009:324:1210–1213. 10.1126/science.1170995 19478186

[93] Schirmer M et al Insight into biases and sequencing errors for amplicon sequencing with the illumina MiSeq platform. Nucleic Acids Res. 2015:43:e37–e37. 10.1093/nar/gku1341 25586220 PMC4381044

[94] Felsenstein J . Confidence limits on phylogenies: an approach using the bootstrap. Evolution. 1985:39:783–791. 10.1111/j.1558-5646.1985.tb00420.x 28561359

[95] Yang Z . On the best evolutionary rate for phylogenetic analysis. Syst Biol. 1998:47:125–133. 10.1080/106351598261067 12064232

[96] Rokas A, Williams BL, King N, Carroll SB. Genome-scale approaches to resolving incongruence in molecular phylogenies. Nature. 2003:425:798–804. 10.1038/nature02053 14574403

[97] Townsend JP . Profiling phylogenetic informativeness. Syst Biol. 2007:56:222–231. 10.1080/10635150701311362 17464879

[98] Philippe H et al Resolving difficult phylogenetic questions: why more sequences are not enough. PLoS Biol. 2011:9:e1000602. 10.1371/journal.pbio.1000602 21423652 PMC3057953

[99] Kumar S, Stecher G, Peterson D, Tamura K. MEGA-CC: computing core of molecular evolutionary genetics analysis program for automated and iterative data analysis. Bioinformatics. 2012:28:2685–2686. 10.1093/bioinformatics/bts507 22923298 PMC3467750

[100] Pavlicev M et al Single-cell transcriptomics of the human placenta: inferring the cell communication network of the maternal-fetal interface. Genome Res. 2017:27:349–361. 10.1101/gr.207597.116 28174237 PMC5340963

[101] Schneider VA et al Evaluation of GRCh38 and de novo haploid genome assemblies demonstrates the enduring quality of the reference assembly. Genome Res. 2017:27:849–864. 10.1101/gr.213611.116 28396521 PMC5411779

[102] Li H, Durbin R. Fast and accurate short read alignment with Burrows-Wheeler transform. Bioinformatics. 2009:25:1754–1760. 10.1093/bioinformatics/btp324 19451168 PMC2705234

[103] Bolger AM, Lohse M, Usadel B. Trimmomatic: a flexible trimmer for illumina sequence data. Bioinformatics. 2014:30:2114–2120. 10.1093/bioinformatics/btu170 24695404 PMC4103590

[104] Saitou N, Nei M. The neighbor-joining method: a new method for reconstructing phylogenetic trees. Mol Biol Evol. 1987:4:406–425. 10.1093/oxfordjournals.molbev.a040454 3447015

[105] Felsenstein J . PHYLIP—phylogeny inference package (version 3.2). Cladistics. 1989:5:164–166.

[106] R Core Team . R: a language and environment for statistical computing https://www.R-project.org

[107] Blondel VD, Guillaume J-L, Lambiotte R, Lefebvre E. Fast unfolding of communities in large networks. J Stat Mech. 2008:10:P10008. 10.1088/1742-5468/2008/10/P10008

[108] Trapnell C et al The dynamics and regulators of cell fate decisions are revealed by pseudotemporal ordering of single cells. Nat Biotechnol. 2014:32:381–386. 10.1038/nbt.2859 24658644 PMC4122333

[109] Qiu X et al Reversed graph embedding resolves complex single-cell trajectories. Nat Methods. 2017:14:979–982. 10.1038/nmeth.4402 28825705 PMC5764547

[110] Cao J et al The single-cell transcriptional landscape of mammalian organogenesis. Nature. 2019:566:496–502. 10.1038/s41586-019-0969-x 30787437 PMC6434952

[111] Wolfram Research Company . Mathematica. Wolfram Research, Inc.; 2020.

[112] Polly PD . Phylogenetics for Mathematica. Version 6.5. Indiana University; 2019. Department of Earth and Atmospheric Sciences.

[113] Zachar, I. 2017, IstvanZachar/Phylogenetics. GitHub repository, GitHub, pp. 686980ed686781c686980dcd686982ff111438bb686935a686986c686983a686951f686982b686984.

[114] Xu Z, Hao B. CVTree update: a newly designed phylogenetic study platform using composition vectors and whole genomes. Nucleic Acids Res. 2009:37:W174–W178. 10.1093/nar/gkp278 19398429 PMC2703908

[115] Quek S-P et al Dissecting comimetic radiations in heliconius reveals divergent histories of convergent butterflies. Proc Natl Acad Sci U S A. 2010:107:7365–7370. 10.1073/pnas.0911572107 20368448 PMC2867687

[116] Schwery O, O’Meara BC. MonoPhy: a simple R package to find and visualize monophyly issues. PeerJ. 2016:4:e1446.

[117] Gusfield D . Algorithms on strings, trees, and sequences: computer science and computational biology. Cambridge University Press; 1997.

[118] Steel M . Phylogeny: discrete and random processes in evolution. Society for Industrial and Applied Mathematics; 2016.

[119] Hicks SC, Townes FW, Teng M, Irizarry RA. Missing data and technical variability in single-cell RNA-sequencing experiments. Biostatistics. 2018:19:562–578. 10.1093/biostatistics/kxx053 29121214 PMC6215955

[120] Kimura M . A simple method for estimating evolutionary rates of base substitutions through comparative studies of nucleotide sequences. J Mol Evol. 1980:16:111–120. 10.1007/BF01731581 7463489

[121] Gawad C, Koh W, Quake SR. Single-cell genome sequencing: current state of the science. Nat Rev Genet. 2016:17:175–188. 10.1038/nrg.2015.16 26806412

[122] Zafar H, Wang Y, Nakhleh L, Navin N, Chen K. Monovar: single-nucleotide variant detection in single cells. Nat Methods. 2016:13:505–507. 10.1038/nmeth.3835 27088313 PMC4887298

[123] Martincorena I et al High burden and pervasive positive selection of somatic mutations in normal human skin. Science. 2015:348:880–886. 10.1126/science.aaa6806 25999502 PMC4471149

[124] Li Y et al Single-cell sequencing analysis characterizes common and cell-lineage-specific mutations in a muscle-invasive bladder cancer. Gigascience. 2012:1:12. 10.1186/2047-217X-1-12 23587365 PMC3626503

[125] Knöfler M et al Human placenta and trophoblast development: key molecular mechanisms and model systems. Cell Mol Life Sci. 2019:76:3479–3496. 10.1007/s00018-019-03104-6 31049600 PMC6697717

[126] Huppertz B, Gauster M. Trophoblast fusion and differentiation: villous cytotrophoblasts and their syncytial derivatives. Hum Reprod Update. 2021:27:904–922. 10.1093/humupd/dmab015 34125187

[127] Yang Z . Computational molecular evolution. Oxford University Press; 2006.

[128] Lewis PO . A likelihood approach to estimating phylogeny from discrete morphological character data. Syst Biol. 2001:50:913–925. 10.1080/106351501753462876 12116640

[129] Tate JG et al COSMIC: the catalogue of somatic mutations in cancer. Nucleic Acids Res. 2019:47:D941–d947. 10.1093/nar/gky1015 30371878 PMC6323903

[130] Kim SN et al Cell lineage analysis with somatic mutations reveals late divergence of neuronal cell types and cortical areas in human cerebral cortex [preprint]. bioRxiv 2023;565899. 10.1101/2023.11.06.565899

[131] Phipson B, Zappia L, Oshlack A. Gene length and detection bias in single cell RNA sequencing protocols. F1000Res. 2017:6:595. 10.12688/f1000research.11290.1 28529717 PMC5428526

[132] Lake JA . Reconstructing evolutionary trees from DNA and protein sequences: paralinear distances. Proc Natl Acad Sci U S A. 1994:91:1455–1459. 10.1073/pnas.91.4.1455 8108430 PMC43178

[133] Muyas F et al De novo detection of somatic mutations in high-throughput single-cell profiling data sets. Nat Biotechnol. 2024:42:758–767. 10.1038/s41587-023-01863-z 37414936 PMC11098751

[134] Shiau C-K et al High throughput single cell long-read sequencing analyses of same-cell genotypes and phenotypes in human tumors. Nat Commun. 2023:14:4124. 10.1038/s41467-023-39813-7 37433798 PMC10336110

[135] Ortega-Batista A et al Single-cell sequencing: genomic and transcriptomic approaches in cancer cell biology. Int J Mol Sci. 2025:26:2074. 10.3390/ijms26052074 40076700 PMC11901077

[136] Kivioja T et al Counting absolute numbers of molecules using unique molecular identifiers. Nat Methods. 2011:9:72–74. 10.1038/nmeth.1778 22101854

[137] Kennedy SR et al Detecting ultralow-frequency mutations by Duplex sequencing. Nat Protoc. 2014:9:2586–2606. 10.1038/nprot.2014.170 25299156 PMC4271547

[138] La Manno G et al RNA velocity of single cells. Nature. 2018:560:494–498. 10.1038/s41586-018-0414-6 30089906 PMC6130801

