# Supplementary material for: Reconstruction of cell diversity and cell lineages from somatic mutations in single-cell transcriptomic data
Source: DNA Res. 2026 Aug 25;33(4):dsag007. doi: 10.1093/dnares/dsag007 (PMC13504284; doi:10.1093/dnares/dsag007)
Supplement: Supplementary file 1 [file dsag007_supplementary_data.docx]

**Supplementary figure 1**

A model system integrating the role of Notch/Wnt signaling in trophoblast stemness and extravillous trophoblast differentiation ^1^. vCTB: villous cytotrophoblast including intravillous cytotrophobrlasts (CYT1, CYT2, and CYT3); CCT: cell column trophoblast; EVT: extravillous trophoblast; N1^ICD^: Notch1 intracellular domain; TEAD4: transcriptional enhancer factor TEF-3 gene; p63: tumor protein p63; TCF1: transcription factor T cell factor 1; TCF4: transcription factor T cell factor 4; IRF6: interferon regulatory factor 6; NOTCH2: notch receptor 2. Note that the terms, CYT and vCTB, are often used interchangeably, as they refer to the same cell population found within the chorionic villi of the placenta ^2^.

**Supplementary figure 2**

The pipeline used in this study for detecting somatic mutations from single-cell data. FASTQ ^3^, BAM ^4^ , and VCF ^5^ files are generated for each single cell.

**Supplementary figure 3**

Examples of variant sites as allelic deviations from the reference genome sequence. Cell 1 and Cell 2: Portions of the read sequences in Cell 1 and Cell 2, respectively. T/T: a homozygous allele in Cell 1; C/T: two heterozygous alleles in Cell 2. In this step, we simply select all sites with any alleles deviated from the reference genome.

**Supplementary figure 4**

Distribution of genome coverage per cell based on the mapping of transcriptome data onto the human reference genome (GRCh38.104). The x-axis shows the percentage of the genome covered by mapped RNA-seq reads for each cell, reflecting the breadth of transcriptome representation. The dataset comprises single-cell transcriptomes collected from two pregnant women, with each bar representing one cell. Red bars indicate cells with discrepant classification between gene expression-based clustering and the RTCA-derived lineage (see Figures 6 and 7), suggesting possible misclassification or ambiguous cell identity**.**

**Supplementary Table 1. Correspondences between single cell ID (Batch 1), Batch cell ID, and GSM ID.** Note: Batch cell IDs are used to identify the corresponding cluster numbers.

| Single cell ID | Batch cell ID | GSM |
| --- | --- | --- |
| SRR4371525 | B1_Cell_1 | GSM2339239 |
| SRR4371527 | B1_Cell_2 | GSM2339240 |
| SRR4371529 | B1_Cell_3 | GSM2339241 |
| SRR4371530 | B1_Cell_4 | GSM2339242 |
| SRR4371531 | B1_Cell_5 | GSM2339243 |
| SRR4371532 | B1_Cell_6 | GSM2339244 |
| SRR4371533 | B1_Cell_7 | GSM2339245 |
| SRR4371534 | B1_Cell_8 | GSM2339246 |
| SRR4371535 | B1_Cell_9 | GSM2339247 |
| SRR4371536 | B1_Cell_10 | GSM2339248 |
| SRR4371537 | B1_Cell_11 | GSM2339249 |
| SRR4371538 | B1_Cell_12 | GSM2339250 |
| SRR4371539 | B1_Cell_13 | GSM2339251 |
| SRR4371540 | B1_Cell_14 | GSM2339252 |
| SRR4371541 | B1_Cell_15 | GSM2339253 |
| SRR4371542 | B1_Cell_16 | GSM2339254 |
| SRR4371543 | B1_Cell_17 | GSM2339255 |
| SRR4371544 | B1_Cell_18 | GSM2339256 |
| SRR4371545 | B1_Cell_19 | GSM2339257 |
| SRR4371546 | B1_Cell_20 | GSM2339258 |
| SRR4371547 | B1_Cell_21 | GSM2339259 |
| SRR4371548 | B1_Cell_22 | GSM2339260 |
| SRR4371549 | B1_Cell_23 | GSM2339261 |
| SRR4371550 | B1_Cell_24 | GSM2339262 |
| SRR4371551 | B1_Cell_25 | GSM2339263 |
| SRR4371552 | B1_Cell_26 | GSM2339264 |
| SRR4371553 | B1_Cell_27 | GSM2339265 |
| SRR4371554 | B1_Cell_28 | GSM2339266 |
| SRR4371555 | B1_Cell_29 | GSM2339267 |
| SRR4371556 | B1_Cell_30 | GSM2339268 |
| SRR4371557 | B1_Cell_31 | GSM2339269 |
| SRR4371558 | B1_Cell_32 | GSM2339270 |
| SRR4371559 | B1_Cell_33 | GSM2339272 |
| SRR4371560 | B1_Cell_34 | GSM2339275 |
| SRR4371561 | B1_Cell_35 | GSM2339277 |
| SRR4371562 | B1_Cell_36 | GSM2339280 |
| SRR4371563 | B1_Cell_37 | GSM2339281 |
| SRR4371564 | B1_Cell_38 | GSM2339282 |
| SRR4371565 | B1_Cell_39 | GSM2339283 |
| SRR4371566 | B1_Cell_40 | GSM2339284 |
| SRR4371567 | B1_Cell_41 | GSM2339285 |
| SRR4371568 | B1_Cell_42 | GSM2339286 |
| SRR4371569 | B1_Cell_43 | GSM2339287 |
| SRR4371570 | B1_Cell_44 | GSM2339288 |
| SRR4371571 | B1_Cell_45 | GSM2339289 |
| SRR4371572 | B1_Cell_46 | GSM2339290 |
| SRR4371573 | B1_Cell_47 | GSM2339291 |
| SRR4371574 | B1_Cell_48 | GSM2339292 |
| SRR4371575 | B1_Cell_49 | GSM2339293 |
| SRR4371576 | B1_Cell_50 | GSM2339294 |
| SRR4371577 | B1_Cell_51 | GSM2339295 |
| SRR4371578 | B1_Cell_52 | GSM2339296 |
| SRR4371579 | B1_Cell_53 | GSM2339297 |
| SRR4371580 | B1_Cell_54 | GSM2339298 |

**Supplementary Table 2. Correspondence between single cell ID (Batch 2), Batch cell ID, and GSM ID.** Note: Batch cell IDs are used to identify the corresponding cluster numbers.

| Single cell ID | Batch cell ID | GSM |
| --- | --- | --- |
| SRR4371581 | B2_Cell_1 | GSM2339299 |
| SRR4371582 | B2_Cell_2 | GSM2339300 |
| SRR4371583 | B2_Cell_3 | GSM2339301 |
| SRR4371584 | B2_Cell_4 | GSM2339302 |
| SRR4371585 | B2_Cell_5 | GSM2339303 |
| SRR4371586 | B2_Cell_6 | GSM2339304 |
| SRR4371587 | B2_Cell_7 | GSM2339305 |
| SRR4371588 | B2_Cell_8 | GSM2339306 |
| SRR4371589 | B2_Cell_9 | GSM2339307 |
| SRR4371590 | B2_Cell_10 | GSM2339308 |
| SRR4371591 | B2_Cell_11 | GSM2339309 |
| SRR4371592 | B2_Cell_12 | GSM2339310 |
| SRR4371593 | B2_Cell_13 | GSM2339311 |
| SRR4371594 | B2_Cell_14 | GSM2339312 |
| SRR4371595 | B2_Cell_15 | GSM2339313 |
| SRR4371596 | B2_Cell_16 | GSM2339314 |
| SRR4371597 | B2_Cell_17 | GSM2339315 |
| SRR4371598 | B2_Cell_18 | GSM2339316 |
| SRR4371599 | B2_Cell_19 | GSM2339317 |
| SRR4371600 | B2_Cell_20 | GSM2339318 |
| SRR4371601 | B2_Cell_21 | GSM2339319 |
| SRR4371602 | B2_Cell_22 | GSM2339320 |
| SRR4371603 | B2_Cell_23 | GSM2339321 |
| SRR4371604 | B2_Cell_24 | GSM2339322 |
| SRR4371605 | B2_Cell_25 | GSM2339323 |
| SRR4371606 | B2_Cell_26 | GSM2339324 |
| SRR4371607 | B2_Cell_27 | GSM2339325 |
| SRR4371608 | B2_Cell_28 | GSM2339326 |
| SRR4371609 | B2_Cell_29 | GSM2339327 |
| SRR4371610 | B2_Cell_30 | GSM2339328 |
| SRR4371611 | B2_Cell_31 | GSM2339329 |
| SRR4371612 | B2_Cell_32 | GSM2339330 |
| SRR4371613 | B2_Cell_33 | GSM2339331 |

**Cell Population Kinetics**

Total mitoses in cell-population kinetics ^6^ can be expressed as

$$\text{Total mitoses}=\int_{0}^{D} \frac{p\left( t \right) N\left( t \right)}{T\left( t \right)} dt\approx\frac{p N_{\text{avg}}}{T} D \text{and add net growth }\left( \approx N_{\text{final}} \right),$$

where $t$ is time since implantation, $D$ is the duration of active placentation (days; approximately implantation to delivery), $N(t)$ is the total number of placental cells at time $t$ (equivalently, the size of the renewing compartment), $p(t)$ is the proliferative fraction at time $t$ (fraction of cells in cycle, often estimated from Ki-67/BrdU labeling), $T\left( t \right)$ is the mean cell-cycle time of proliferating cells at time t (days per division), $N_{\text{avg}}$ is the time-average of $N(t)$ over [0, D], $p$ and $T$ (without time dependence) are time-averaged values of $p(t)$ and $T\left( t \right)$, respectively, and $N_{\text{final}}$ is the final placental cell count at term.

Assuming $p \gtrsim20\%$, $T \lesssim0.5\text{-}0.75\text{d}$, and $N_{\text{avg}} \sim0.5 N_{\text{final}}$ based on placental turnover studies ^7-9^, a plausible upper bound for total mitoses is on the order of $\sim2\text{-}3 \times{10}^{13}$.

**Formalization of variant patterns in scRNA‐seq data**

In scRNA‐seq data, the observed “variant pattern” depends not only on the underlying genomic genotype but also on the stochastic nature of transcription and technical factors (such as allelic dropout). Under the same biological assumptions (at most only one new mutation per site; mutations can be germline—either inherited or de novo—or somatic), a diploid cell’s genome can be in one of the two states at a given site:

**• Homozygous reference:** $\left( R,R \right)\mathbf{,}$

**• Heterozygous:** $\mathbf{(}R,V)$,

where $R$ and $V$ represent the reference and variant alleles, respectively. Although another genotype is possible (Homozygous variant: $\mathbf{(}V,V)$), it can be disregarded under the assumption that at most only one new mutation occurs per site.

Because scRNA‐seq measures expressed transcripts rather than the full genome, the observed transcript set may not directly reflect the underlying genotype. Let $T(c)$ denote the set of alleles observed in the transcripts of cell $c$ at the variant site. The distinct patterns we might observe are:

### 1. Only the reference allele is observed: $\boldsymbol{T(c)=\{R\}}$

- **Possible underlying genotypes are:**
  - **Homozygous reference (**$(R,R)$**)** – all transcripts should be $R$.
  - **Heterozygous (**$(R,V)$**) with monoallelic expression or allelic dropout:** Even though the cell carries both alleles, only the reference allele is captured (or expressed) in that particular cell.

### 2. Only the variant allele is observed: $\boldsymbol{T(c)=\{V\}}$

- **Possible underlying genotypes are:**
  - **Homozygous variant (**$(V,V)$**)** – all transcripts are $V$ (This genotype is ignored because of the above assumption).
  - **Heterozygous (**$(R,V)$) **with monoallelic expression or allelic dropout:** In this case, only the variant allele is observed due to transcriptional bias or dropout of the reference allele.

### 3. Both the reference and variant alleles are observed: $\boldsymbol{T(c)=\{R,V\}}$

In an ideal, unbiased situation, the expression ratio is likely ~1:1, reflecting equal expression of the $R$ and $V$ alleles; however, due to technical and biological variability, this ratio can deviate from 1:1.

In the presence of **germline mutations (inherited or de novo),** all cells harbor the mutation. For a heterozygous germline variant, every cell’s genomic state is $\{R,V\}$; yet, in the transcriptome:

- Some cells may show $\{R,V\}$ (if both alleles are expressed)
- Others may show $\{R\}$ or $\{V\}$ (if monoallelic expression or dropout occurs).

When we have **somatic (mosaic) mutations,** only a subset of cells carry the mutation (they are typically heterozygous, $(R,V)$). In affected cells, the same transcriptome outcomes as above are possible:

- $\{R,V\}$ when both alleles are expressed,
- $\{R\}$ or $\{V\}$ if only one allele is missed.

For cells without the mutation (or cells where the mutation is absent in the case of mosaicism), we observe only the reference transcripts $\{R\}$.Therefore, the detection of somatic mutations can be formalized as follows using first-order predicate logic symbols ^10-13^:

Underlying genotypes: each diploid cell has one of the following two genotypes:

$$g(c)\in\{(R,R),(R,V)\}$$

where $R$ is the reference allele and $V$ is the variant allele. Note that genotype $(V,V)$ is disregarded because of the assumption.

**Observed transcript patterns:** Because of stochastic gene expression, the observed set of alleles in a cell (denoted by $O(c)$) may be:

- $\{R\}$ (only the reference allele detected),
- $\{V\}$ (only the variant detected), or
- $\{R,V\}$ (both alleles detected).

In an ideal scenario, a heterozygous cell would yield $\{R,V\}$; however, allelic dropout or monoallelic expression can lead to an observation of only one allele.

**Somatic mutation first-order predicate logic:** A somatic mutation is expected to be present only in a subset of cells. Formally, if we define:

- $\mathrm{Som}(m)$ denotes that mutation m is a somatic mutation, and
- $O\left( c,a \right)$ denotes that in cell $c$, the allele type a is observed, where a is either $R$ or $V$,

then the logical condition for a somatic mutation is:

$\mathrm{Som}(m)\to(\exists c_{1},(O(c_{1},V)\wedge\neg O(c_{1},R))\wedge\exists c_{2},(O(c_{2},R)\wedge\neg O(c_{2},V)\wedge c_{1}\neq c_{2}))$,

where $\exists$, $\wedge$, $\neg$ denote “there exists”, “and”, and “not”, respectively. In other words, if $m$ is a somatic mutation, there exists a cell $c_{1}$ where the variant is observed at the site but the reference is not, and there exists a different cell $c_{2}$ where the reference is observed at the site but the variant is not.

In a mosaic scenario ^14^ (genetic mosaicism), at least one cell shows evidence of the variant while at least another does not. For a germline mutation (whether inherited or de novo in the germline), the variant would be observed consistently across nearly all cells (modulo dropout: i.e., missing data or gene expression due to stochastic sampling issues).

To distinguish somatic from germline variants, we need to perform cross-cell comparisons: i.e., to consider the variant calls across cells. For a true germline mutation, the variant will appear in every cell (except for dropouts), whereas a somatic mutation will display a mosaic pattern where at least one cell shows evidence of the variant whereas at least another cell does not:

- Germline**:**

$$\forall c,g(c)\in\{(R,V)\}$$

- Somatic**:**

$\exists c_{1},g\left( c_{1} \right)\in\left\{ \left( R,V \right) \right\}$ and $\exists c_{2},g(c_{2})=(R,R)$

Note that genotype $\left( V,V \right)$ is disregarded because of the assumption.

Now we propose the following theorem:

**Theorem:** Assume that at most one new (de novo) mutation can occur per site in the germline. Then, at least one somatic mutation should have occurred at a tri-allelic variant site.

**Definitions:**

**Diploidy:** Each cell $c$ has a genotype

$$g(c)\in\{(a,b)\mid a,b\in\mathcal{A}\}$$

where $\mathcal{A}$ is the set of all possible alleles and, by definition, $|\{a,b\}|\leq2$.

**Germline mutations:** If a mutation is germline (inherited or de novo in the germline), then it is present in all cells. In any cell, the genotype will be either homozygous or heterozygous for a pair of alleles.

**Somatic mutations:** A somatic mutation is found in only a subset of cells. In such cells, one allele may have been altered relative to the germline state, potentially creating a new allele not present in all cells.

**Observation:** At a particular genomic site, we observe a tri-allelic pattern in a collection of cells. That is, the set of alleles observed is:

$A_{\text{obs}}=\{a,b,c\}$ with $|A_{\mathrm{obs}}|=3$

**Proof:** Suppose that no **somatic mutation** occurs at the site and we observe a tri-allelic variant site. Every mutation event is either an inherited germline variant or a de novo **germline mutation**. By the assumption of **diploidy** and the assumption of at most one de novo mutation per site, every cell $c$ will have a genotype

$g(c)\in\{(a,a),(a,b),(b,b)\}$,

where $\{a,b\}\subset\mathcal{A}$ and $|\{a,b\}|\leq2$. Since every cell carries only alleles from the set $\{a,b\}$, the aggregate set of observed alleles across all cells will satisfy:

$A_{\mathrm{obs}}\subseteq\{a,b\}$.

Therefore, $|A_{\mathrm{obs}}|\leq2$. However, we are given that $|A_{\mathrm{obs}}|=3$ (i.e., the variant site is tri-allelic). This contradicts the conclusion of $|A_{\mathrm{obs}}|\leq2$. That is, the assumption that no somatic mutation occurs leads to a contradiction. Therefore, to explain the presence of three distinct alleles, at least one somatic mutation must have occurred. In other words, the third allele can only be introduced by a mutation that arose in a subset of cells (i.e., a somatic event).

**Derivation of Eqs. (16) and (17)**

Let $\mu_{\text{gen}}$denote the genome-wide somatic mutation rate per cell division (units: SNVs/division across the whole genome of $3\times{10}^{9}$bp). Because only the across-cell intersection of callable bases is analyzed, the observable fraction of the genome is

$$f_{\text{obs}}\text{ }=\text{ }\frac{L_{\text{align}}}{3\times{10}^{9}}.$$

Under the neutral infinite-sites model, the expected number of observed segregating sites in a sample of $n$ cells is the product of the mutation rate ($\text{ }\mu_{\text{gen}}$), the total tree length (in divisions), and the observable fraction is

$$\mathbb{E}[S]\text{ }=\text{ }\mu_{\text{gen}}\text{ }\text{ }\mathbb{E}[T_{n}]\text{ }\text{ }f_{\text{obs}}\text{ }=\text{ }\mu_{\text{gen}}\text{ }\mathbb{E}[T_{n}]\text{ }\frac{L_{\text{align}}}{3\times{10}^{9}}.$$

Solving for the required genome-wide per-division rate that yields a target $S$ gives

$$r_{\text{req}} = \mu_{\text{gen}}= \frac{S}{E\left[ T_{n} \right]} \frac{3\times{10}^{9}}{L_{\text{align}}}\quad\text{(SNVs}\text{/}\text{division).}$$

Note that the unit of $r_{\text{req}}$ is $[S]/[\text{divisions}]\times[\text{bp}]/[\text{bp}]=\text{SNVs}/\text{division}$.

The corresponding per-cell burden at term (SNVs per genome in a terminal cell) is then

$$M_{\text{cell}}=r_{\text{req}} h\quad\text{(SNVs per cell).}$$

**MEGA X full parameters**

; Instead of modifying this file, simply create a new MEGA Analysis Options file by using MEGA.

[ MEGAinfo ]

ver	= 11220624-x86_64 Linux

[ DataSettings ]

datatype	= snNucleotide

containsCodingNuc	= False

MissingBaseSymbol	= ?

IdenticalBaseSymbol	= .

GapSymbol	= -

Labelled Sites	= All Sites

Labels to Include	=

[ ProcessTypes ]

ppInfer	= true

ppME	= true

[ AnalysisSettings ]

Analysis	= Phylogeny Reconstruction

Scope	= All Selected Taxa

Statistical Method	= Minimum Evolution method

Phylogeny Test	= ====================

Test of Phylogeny	= None

No. of Bootstrap Replications	= Not Applicable

Substitution Model	= ====================

Substitutions Type	= Nucleotide

Model/Method	= Maximum Composite Likelihood

Substitutions to Include	= d: Transitions + Transversions

Rates and Patterns	= ====================

Rates among Sites	= Uniform Rates

Gamma Parameter	= Not Applicable

Pattern among Lineages	= Same (Homogeneous)

Data Subset to Use	= ====================

Gaps/Missing Data Treatment	= Pairwise deletion

Site Coverage Cutoff (%)	= Not Applicable

Tree Inference Options	= ====================

ME Heuristic Method	= Close-Neighbor-Interchange (CNI)

Initial Tree for ME	= Obtain initial tree by Neighbor-Joining

ME Search Level	= 1

System Resource Usage	= ====================

Number of Threads	= 32

Has Time Limit	= False

Maximum Execution Time	= -1

**References**

1.	Knöfler, M., Haider, S., Saleh, L., Pollheimer, J., Gamage, T. K. J. B. and James, J. 2019, Human placenta and trophoblast development: key molecular mechanisms and model systems. *Cellular and Molecular Life Sciences*, **76**, 3479-3496.

2.	Huppertz, B. and Gauster, M. 2021, Trophoblast fusion and differentiation: Villous cytotrophoblasts and their syncytial derivatives. *Human Reproduction Update*, **27**, 904-921.

3.	Cock, P. J. A., Fields, C. J., Goto, N., Heuer, M. L. and Rice, P. M. 2010, The Sanger FASTQ file format for sequences with quality scores, and the Solexa/Illumina FASTQ variants. *Nucleic acids research*, **38**, 1767-1771.

4.	The SAM/BAM Format Specification Working Group 2020, Sequence Alignment/Map Format Specification.

5.	Danecek, P., Auton, A., Abecasis, G., et al. 2011, The variant call format and VCFtools. *Bioinformatics (Oxford, England)*, **27**, 2156-2158.

6.	Steel, G. G. 1977, *Growth Kinetics of Tumours: Cell Population Kinetics in Relation to the Growth and Treatment of Cancer*. Clarendon Press: Oxford.

7.	Mayhew, T. M. 2014, Turnover of human villous trophoblast in normal pregnancy: what do we know and what do we need to know? *Placenta*, **35**, 229-240.

8.	Huppertz, B., Kingdom, J., Caniggia, I., et al. 2003, Hypoxia favours necrotic versus apoptotic shedding of placental syncytiotrophoblast into the maternal circulation. *Placenta*, **24**, 181-190.

9.	Coorens, T. H. H., Oliver, T. R. W., Sanghvi, R., et al. 2021, Inherent mosaicism and extensive mutation of human placentas. *Nature*, **592**, 80-85.

10.	Tuyéras, R. Category Theory for Genetics I: Mutations and Sequence Alignments. arXiv preprint.

11.	Ashburner, M., Ball, C. A., Blake, J. A., et al. 2000, Gene Ontology: tool for the unification of biology. *Nature Genetics*, **25**, 25-29.

12.	1906, (1) Symbolic Logic and its Applications (2) The Development of Symbolic Logic (3) An Introduction to Logic (4) Thought and Things, or Genetic Logic. *Nature*, **75**, 1-2.

13.	Thomas, R. 1973, Boolean formalization of genetic control circuits. *Journal of Theoretical Biology*, **42**, 563-585.

14.	Sun, X.-L., Chen, Z.-H., Guo, X., et al. 2023, Stem cell competition driven by the Axin2-p53 axis controls brain size during murine development. *Developmental Cell*, **58**, 744-759.e711.
